# Supplementary material for: Inferred Attractiveness: A generalized mechanism for sexual selection that can maintain variation in traits and preferences over time
Source: PLoS Biol. 2023 Oct 3;21(10):e3002269. doi: 10.1371/journal.pbio.3002269 (PMC10547189; doi:10.1371/journal.pbio.3002269)
Supplement: S1 Text — To investigate how the interaction of cultural transmission and context dependence differs from either such circumstance in isolation, we include, in addition to our basic model, 3 reference models that consider these 2 processes (cultural transmission and frequency dependence) independently. These are Reference Model 1: Direct Copying of Female Preferences (Cultural Transmission), Reference Model 2: Preference for Male Trait that has the Most Rare Variant, and Preference for a Trait Variant from a Randomly Observed Male (IA without social learning), and Reference Model 3: Preference for Rare/Novel Male Traits and Trait Variants. S1 Text explains the parameters of and major findings from each reference model, and results are shown graphically in S1 Fig. (DOCX) [file pbio.3002269.s001.docx]

### S1 Text. Reference models. To investigate how the interaction of cultural transmission and context dependence differs from either such circumstance in isolation, we include, in addition to our basic model, three reference models that consider these two processes (cultural transmission and frequency dependence) independently.

### Reference Model 1: Direct Copying of Female Preferences (Cultural Transmission)

The first control scenario we model is simply one in which juvenile females acquire their preferences by observing mating decisions of an older female, and then accurately copy those preferences (results in S1 Fig ai-iii). This approach removes the effect of mistaken inference that is a key part of the Inferred Attractiveness (IA) model, but retains the phenomenon of copying. Rather than being influenced by the relative frequencies of the different trait variants, this scenario assumes that juvenile females have un-erring knowledge of the trait category (e.g., color or pattern in S1 Table) on which the observed female bases her choice. In accordance with previous copying models [1, 2], females then prefer the trait variant (e.g., for color, light or dark) of the chosen male.

To model this case, we create a situation where juvenile females observe matings with different types of males based on the proportions of these matings in the population, and assume that juveniles precisely copy (with omniscience of the female’s preference phenotype) the preference at the P trait of the observed females. As with the basic model, a small population of females is created where the number of females of each genotype *i* is $x_{f, i}^{N}$ (see Methods). Each female randomly observes a mated pair, in proportion to their occurrence. The female acquires her P phenotype based on the P phenotype of the female in the pair, and sets the phenotypes at the OA and OB traits by the TA and TB alleles present in the male of the pair.

### Reference Model 2: Preference for Male Trait that has the Most Rare Variant, and Preference for a Trait Variant from a Randomly Observed Male (IA without social learning)

This scenario removes the effect of mate choice copying, but retains the frequency dependence that occurs in IA because of encounter frequencies with different male phenotypes (results in Extended Data S1 Fig bi-iii). We note that this model variant is biologically unrealistic, but serves as a thought experiment for understanding the IA model. Here, juvenile females simply base their preference (P) on whichever trait type (e.g., TA or TB) has the overall rarest allele in the population, across both trait loci, that is, out of $\mathrm{TA}_{1}$, $\mathrm{TA}_{2}$, $\mathrm{TB}_{1}$ and $\mathrm{TB}_{2}$. This matches our assumption in the main model that females base their preference on the trait type that has the rarest variant, but does not involve focusing on a successfully mated male and using his trait variants when assessing rarity (thus it removes a social learning component). Note that the trait type with the rarest variant will also be the one that has the most common trait variant in the population (since one minus the rarest will be the most common). We assume also that at that trait locus the juvenile females prefer an allelic variant of a randomly sampled male – sampled randomly across the whole population, not just across successfully mated males as in the IA model – to set their phenotype at the OA and OB traits. In this way we preserve the feature of setting the preference for trait variants based on a frequency-dependent observation of a male, but again there is no copying of preferences because the male is a random male from the population, not a successfully mated male. We again assume that females only observe a subset of all males in the population, to maintain stochastic effects, as follows.

First, a sample of males of size *n_males_* is chosen from among the males pooled across both age cohorts (young adults and older adults), where males are picked for this subset in proportion to their frequency in the larger population (the subset is chosen by sampling with replacement). Females base their judgment of the frequencies of the trait variants in the population as a whole on the frequencies in this observed subset. Females thus set their preference P_A_ or P_B_ based on the rarest trait variant in this observed sample. The female then samples a random male from the subset that she has observed and sets her OA and OB trait variants to match the alleles that this male has at the TA and TB loci. The consequence of this process is that females generally prefer the most common male trait variant in the population.

### Reference Model 3: Preference for Rare/Novel Male Traits and Trait Variants

In this final scenario, we assume that females have a preference for rarity both at the level of the trait and the trait variant, given their observations of trait variant frequencies across the observed adult male population (not just in successfully mated males; S1 Fig ci-iii). Similar behavioral mechanisms likely underlie preferences for rarity and novelty [3-5], and so we expect that a novelty preference would produce patterns similar to the rarity preference modeled here. As in the previous model (Reference Model 2), we initially sample a random subset of males from the population at large, averaged across both age cohorts, by sampling with replacement from the general population. This represents the male phenotypes observed by a single female. The female then sets her preference at the P trait by whichever type of trait (TA or TB) has the overall rarest allele out of the males in her observed subset. Unlike in Reference Model 2, she also sets her trait variants at the OA and OB phenotypic traits to match the rarer of the two trait variants at each trait. For example, if the order of rarity (most rare to least rare) of trait variance in the subset observed by a female is TB_2_, TA_1_, TA_2_, TB_1_, that female will obtain the phenotype P_B_, OB_2_, and OA_1_ (although as in the other models, OA_1_ will not be expressed).

Exploration of the behavior of the model shows that preference fluctuations stem from the fact that with overlapping generations, a preference for rarity will lead to lag between trait evolution and the preference that is set for a rare trait. This lag stems from three sources. First, there is often a brief lag in the population-wide preference targeting the rarest trait variant, because when there are age cohorts of females, the preferences of young females average with those of the older cohorts to set the population preference. In our model with 3 cohorts this lag was often one generation. Second, there is a lag because once the preference starts applying selection on the trait it can take a few generations for the frequency of the trait to change enough to cross frequencies with the other trait in the system. Finally, we observed a 3-4 generation lag because of a delay between when the trait sets a new preference and when the preference in turn affects the trait. Specifically, after a new population-wide preference is set (in, say, Generation X), females with the new preference must mate with the males that they prefer (in Generation X+1). Male offspring from that mating remain juveniles for one year (in Generation X+2), then express the trait as young adults (in Generation X+3). Only in this final generation do the young adult male frequencies finally affect preference observations.

**S1 Text References**

1. Servedio MR, Kirkpatrick M. The evolution of mate choice copying by indirect selection. The American Naturalist. 1996;148(5):848-67. doi: 10.1086/285959.

2. Kirkpatrick M, Dugatkin LA. Sexual selection and the evolutionary effects of copying mate choice. Behavioral Ecology and Sociobiology. 1994;34(6):443-9. doi: 10.1007/BF00167336.

3. Potter T, Arendt J, Bassar RD, Watson B, Bentzen P, Travis J, et al. Female preference for rare males is maintained by indirect selection in Trinidadian guppies. Science. 2023;380(6642):309-12. doi: doi:10.1126/science.ade5671.

4. Daniel MJ, Koffinas L, Hughes KA. Habituation underpins preference for mates with novel phenotypes in the guppy. Proc Biol Sci. 2019;286(1902):20190435. Epub 2019/05/16. doi: 10.1098/rspb.2019.0435. PubMed PMID: 31088269; PubMed Central PMCID: PMCPMC6532517.

5. Daniel MJ, Koffinas L, Hughes KA. Mating preference for novel phenotypes can be explained by general neophilia in female guppies. The American Naturalist. 2020;196(4):414-28.
