# Supplementary figures and images for: Inferred Attractiveness: A generalized mechanism for sexual selection that can maintain variation in traits and preferences over time
Source: PLoS Biol. 2023 Oct 3;21(10):e3002269. doi: 10.1371/journal.pbio.3002269 (PMC10547189; doi:10.1371/journal.pbio.3002269)

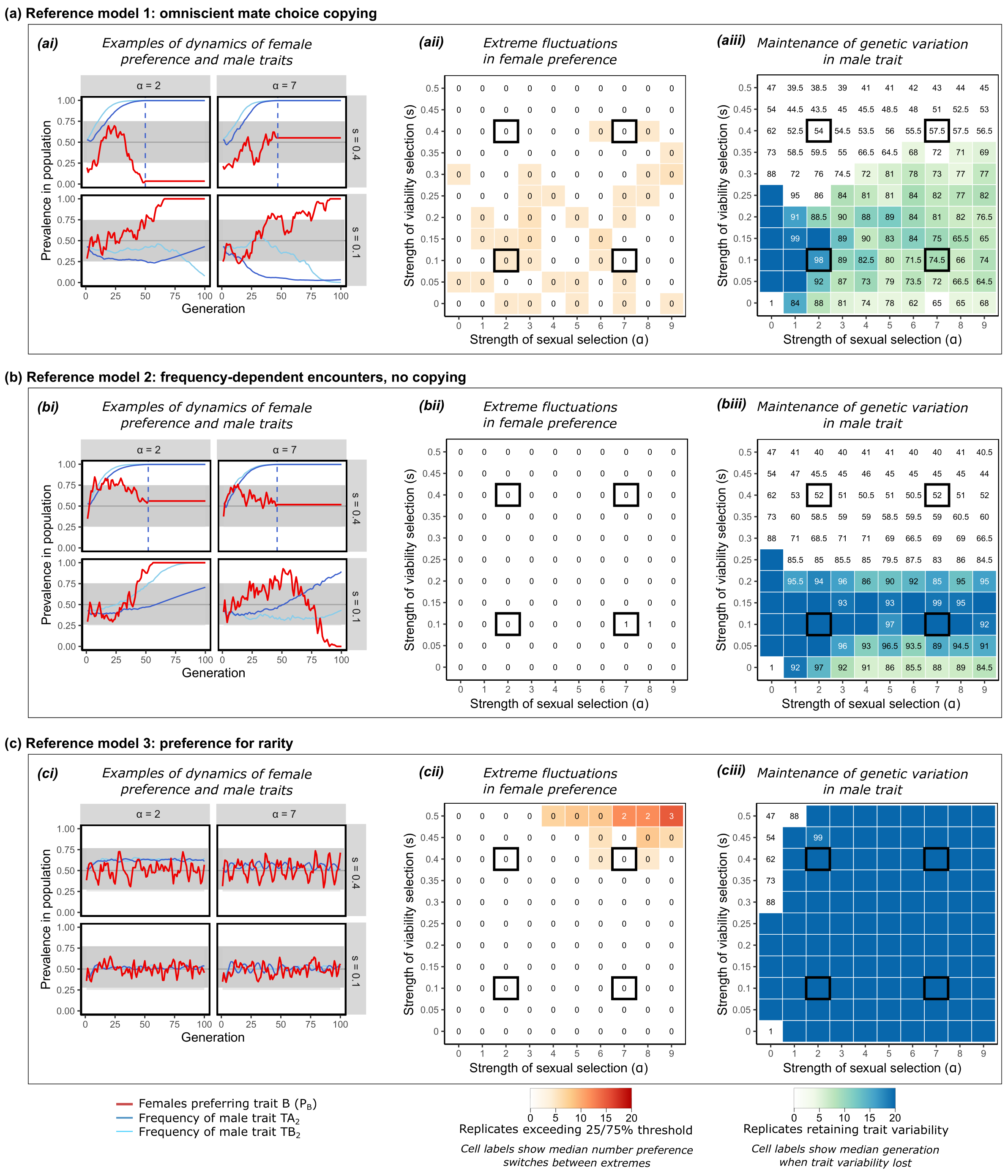

Supplement: S1 Fig — In each case, (i) panels show dynamics of preference and trait variation over time for one exemplar of 20 replicate runs (at parameter values highlighted by bold black boxes in the other plots). Heat maps (panels ii and iii) summarize trends from 20 replicate runs of the model using the conventions established in the figures of the main text. As in previous plots, dashed lines in plots of individual runs (i) indicate the generation in which trait variation is lost. X-axes in (ii) and (iii) define the strength of female preference (α), which is how much more likely a female is to mate with a male that has the trait variant she prefers, relative to a male with the unpreferred variant at that same locus and given that she encounters one of each type. The y-axes in (ii) and (iii) represent the strength of viability selection (s) on allele 2 of each male trait, such that a male with variant 1 is 1-s times less likely to survive than a male with variant 2 of the same trait. Code and raw simulation output used to generate this figure are archived on the Open Science Framework; DOI: 10.17605/OSF.IO/R673J. (TIFF) [file pbio.3002269.s002.tiff]

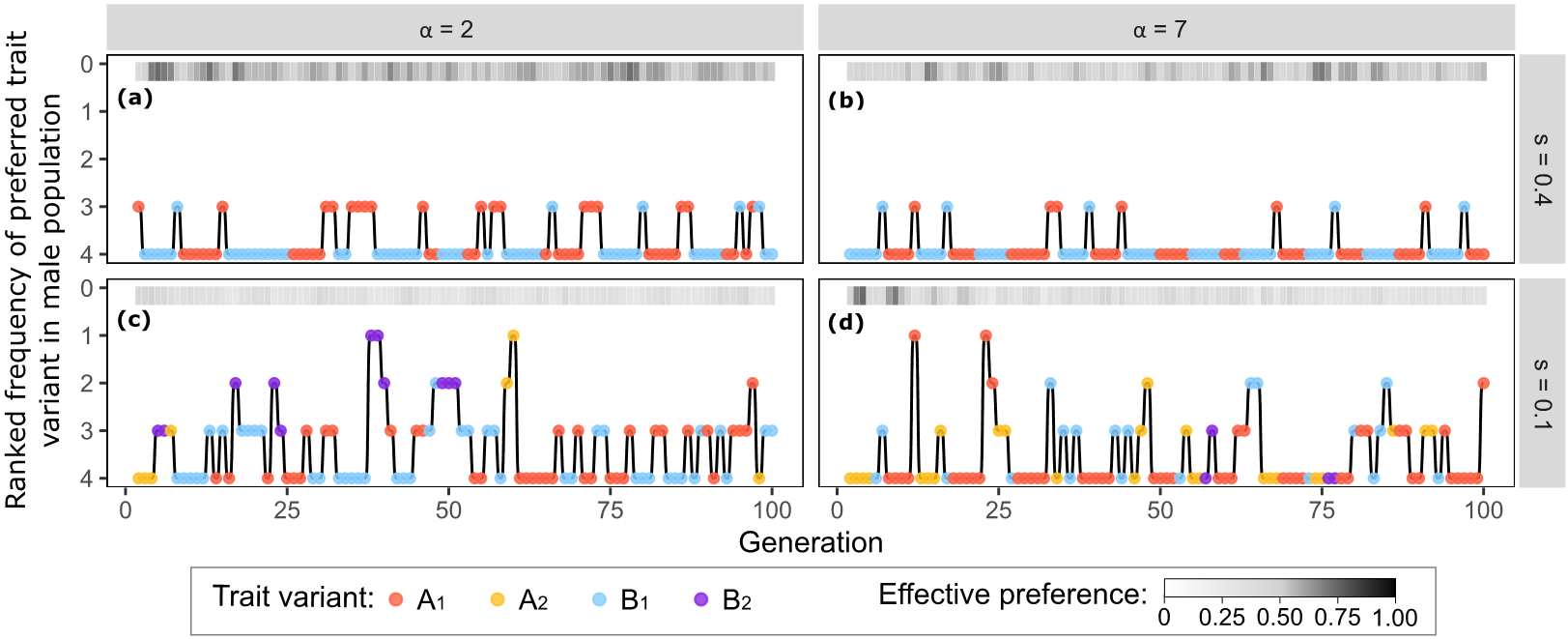

Supplement: S2 Fig — Even when selection favoring some trait variants is extremely strong, females making mate choices by rarity or novelty preference prefer the rare (selectively disfavored) variants. In comparison, mate choice by inferred attractiveness relatively quickly favors selectively advantageous variants when selection is strong (Fig 2). Therefore, although both preference for rarity and IA produce fluctuations in which trait is preferred and maintain variability in mate traits over time, IA does so in a manner that is more responsive to variation in selective strength, via changes in the relative frequency of trait variants. Under a preference for rarity, female preference targets selectively disadvantageous trait variants (A1 or B1) when viability selection on those traits is strong. Panels show generations 2–100 for (a) low sexual selection and high viability selection; (b) high sexual selection and high viability selection; (c) low sexual selection and low viability selection; and (d) high sexual selection and low viability selection (sexual selection strength 0.2 or 0.8; viability selection strength 2 or 7). Y-axes show how common the variants are in the male population ranked from most common (rank 1) to rarest (rank 4), point color indicates which trait variant is most preferred, and the greyscale at the top of the panels indicates the proportion of females expressing preference for the most commonly preferred trait variant. Strength of sexual selection here reflects female preference (α), defined as above as how much more likely a female is to mate with a male that has the trait variant she prefers. Code and raw simulation output used to generate this figure are archived on the Open Science Framework; DOI: 10.17605/OSF.IO/R673J. (TIFF) [file pbio.3002269.s003.tiff]
