# Supplementary material for: Inferred Attractiveness: A generalized mechanism for sexual selection that can maintain variation in traits and preferences over time
Source: PLoS Biol. 2023 Oct 3;21(10):e3002269. doi: 10.1371/journal.pbio.3002269 (PMC10547189; doi:10.1371/journal.pbio.3002269)

Fig S3. Key to plots of exemplar model runs

Page	Model.setting	Male group size	Description	Corres-ponding Figure
S3.i	fd.x	10	Inferred Attractiveness model, with smaller group size, which leads to increased stochasticity of outcomes.	---
S3.ii	fd.x	30	Inferred Attractiveness model, with group size as illustrated in the main text.	1b-d
S3.iii	fd.x	100	Inferred Attractiveness model, with smaller group size, which leads to decreased stochasticity of outcomes.	---
S3.iv	fd.fs	30	Inferred Attractiveness model, with variable viability selection among traits: s_A is held constant at 0.05 while s_B varies from 0 to 0.5.	3a
S3.v	fd.fa	30	Inferred Attractiveness model, with variable sexual selection among traits: α_A is held constant at 2, while α_B varies from 0 to 1.	3b
S3.vi	fde.xpt1	30	Inferred Attractiveness model, with one trait environmentally determined: frequency of TB_2 is reset to 0.1 in each generation.	4
S3.vii	fde.x	30	Inferred Attractiveness model, with one trait environmentally determined at a higher frequency: frequency of TB_2 is reset to 0.4 in each generation, illustrating that patterns generated by the presence of a trait insensitive to selection depend on their frequencies.	---
S3.viii	sc.x	30	Reference model 1: "omniscient" mate choice copying with no neg. frequency-dependence.	S1.a
S3.ix	ncs.x	30	Reference model 2: frequency-dependent choice due to encounter rate which occurs similarly to the AI model, but without social learning of preference.	S1.b
S3.x	pnov.x	30	Reference model 3: rarity/novelty preference	S1.c
S3.xi	pnov.x	100	Reference model 3b: rarity/novelty preference with larger male group sizes, illustrating the larger temporal shifts in population-level female preferences evident with reduced stochasticity of outcomes.	---

Model:fd, setting:x, group size:10 (Preference=red, Trait A=dark blue, Trait B=light blue)

Strength of sexual selection (a) on x−axis (0−9); stength of viability selection (s) panels arrayed on y−axis (0−0.5)

PageS3.i

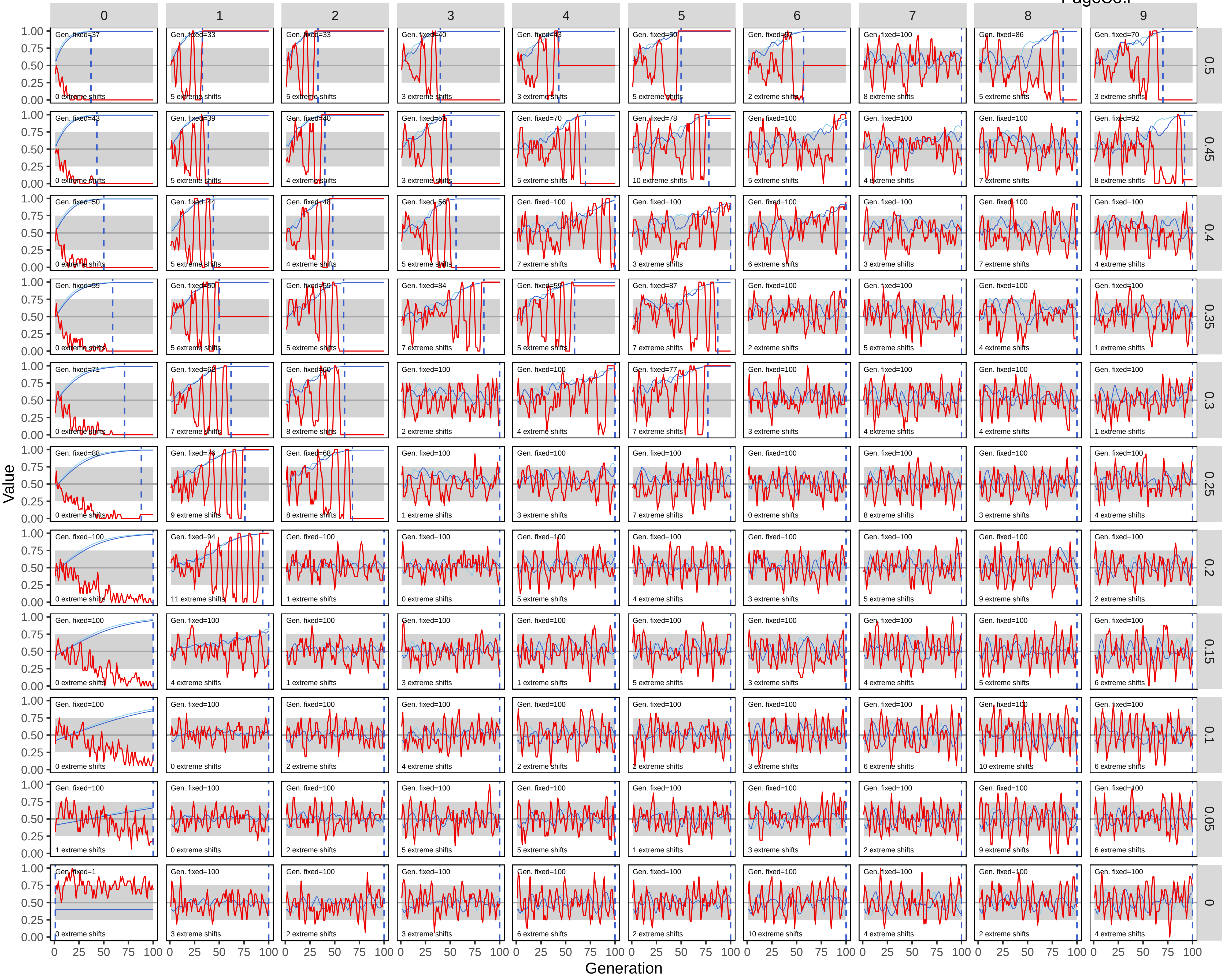

Model:fd, setting:x, group size:30 (Preference=red, Trait A=dark blue, Trait B=light blue)

Strength of sexual selection (a) on x−axis (0−9); strength of viability selection (s) panels arrayed on y−axis (0−0.5)

PageS3.ii

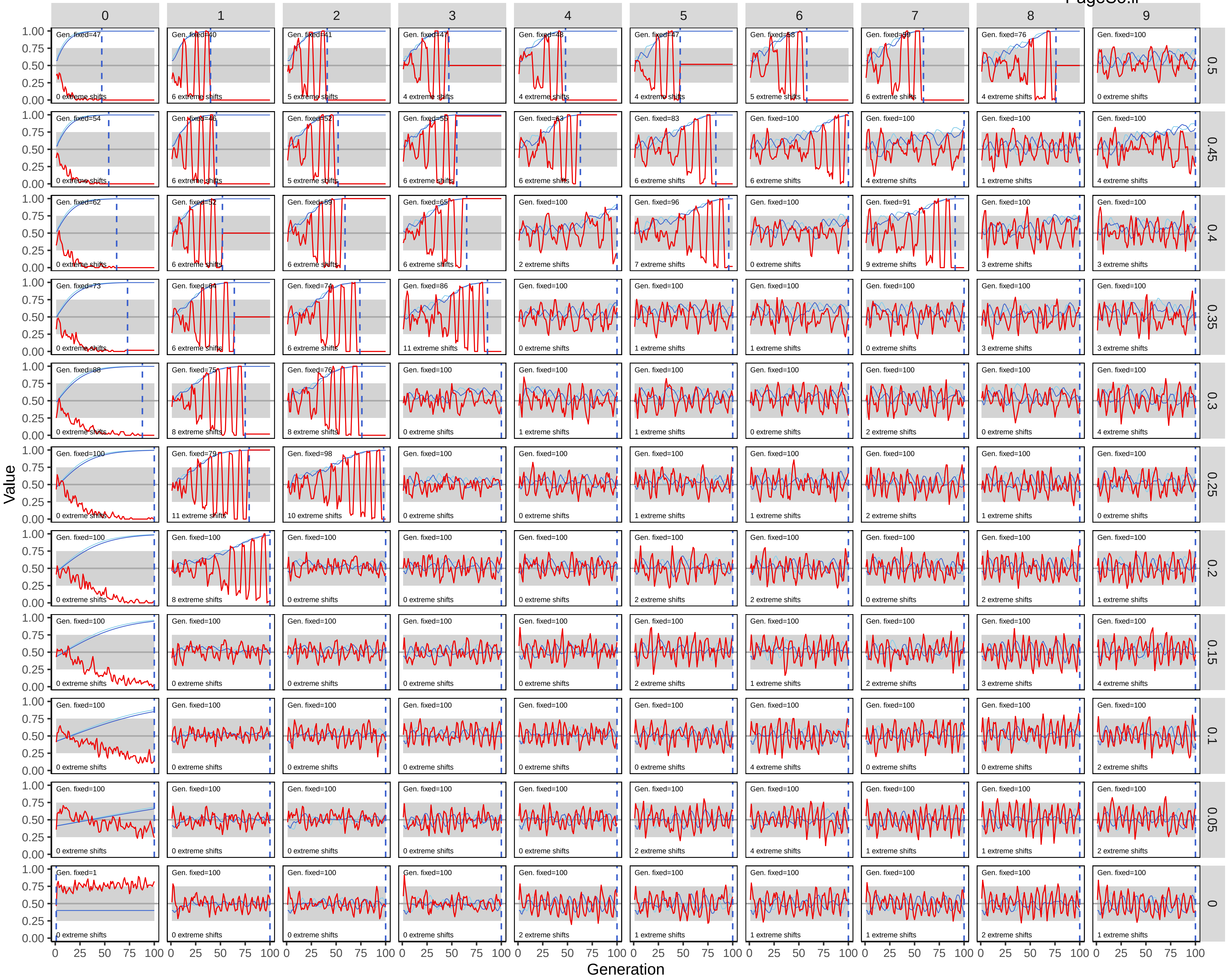

Model:fd, setting:x, group size:100 (Preference=red, Trait A=dark blue, Trait B=light blue)

Strength of sexual selection (a) on x−axis (0−9); stength of viability selection (s) panels arrayed on y−axis (0−0.5)

PageS3.iii

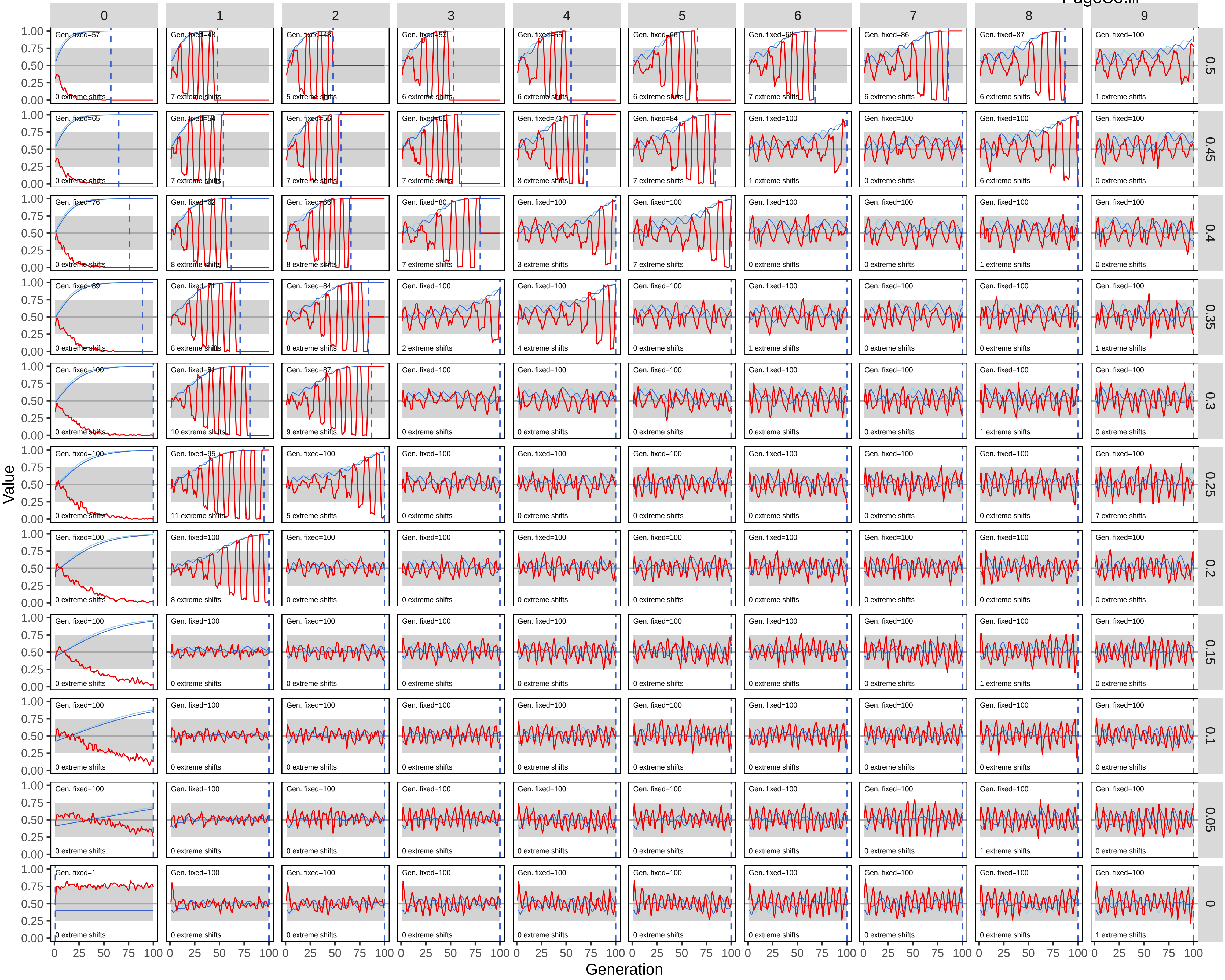

Model:fd, setting:fs, group size:30 (Preference=red, Trait A=dark blue, Trait B=light blue)

Strength of sexual selection (a) on x−axis (0−9); stength of viability selection (s) panels arrayed on y−axis (0−0.5)

PageS3.iv

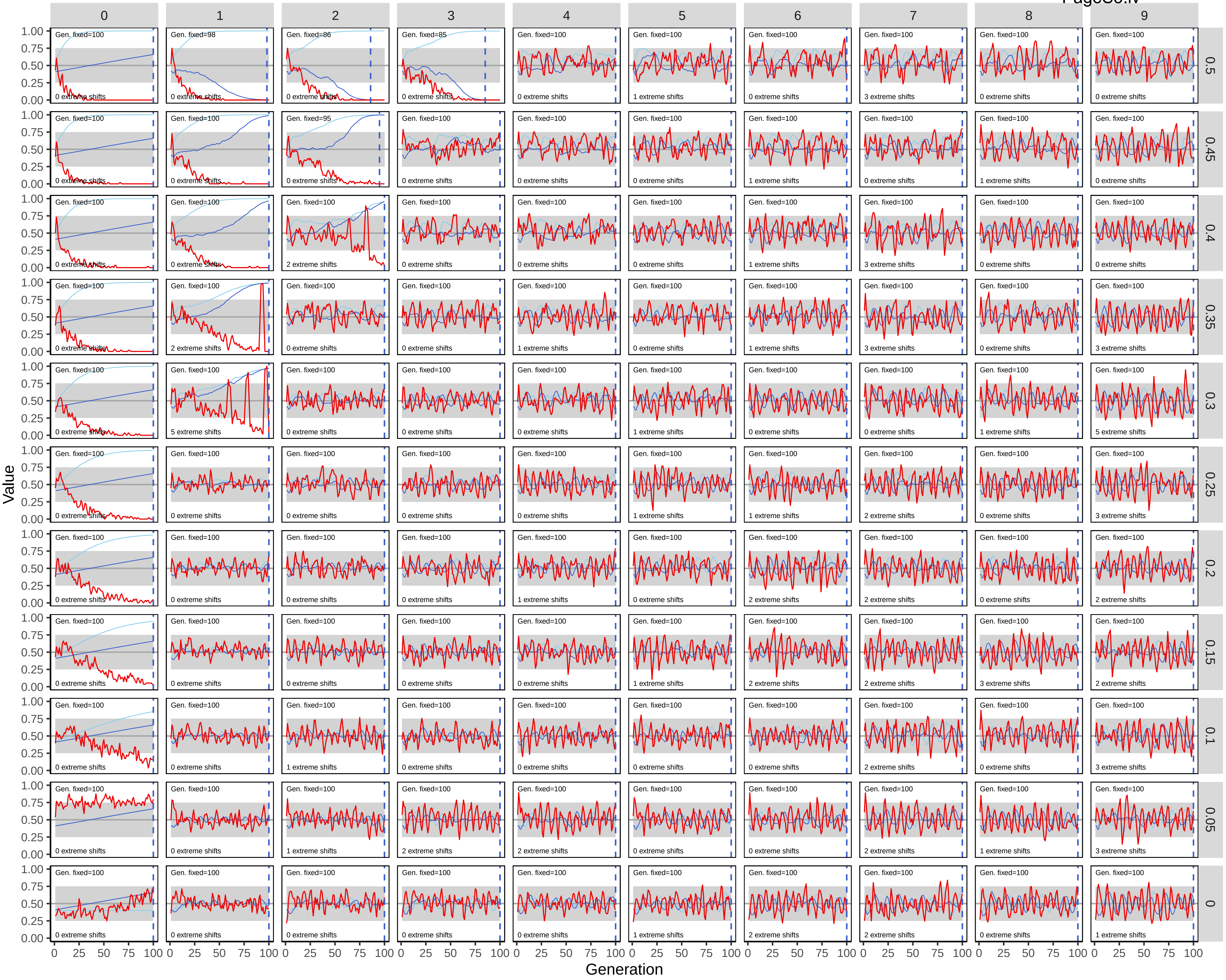

Model:fd, setting:fa, group size:30 (Preference=red, Trait A=dark blue, Trait B=light blue)

Strength of sexual selection (a) on x−axis (0−9); strength of viability selection (s) panels arrayed on y−axis (0−0.5)

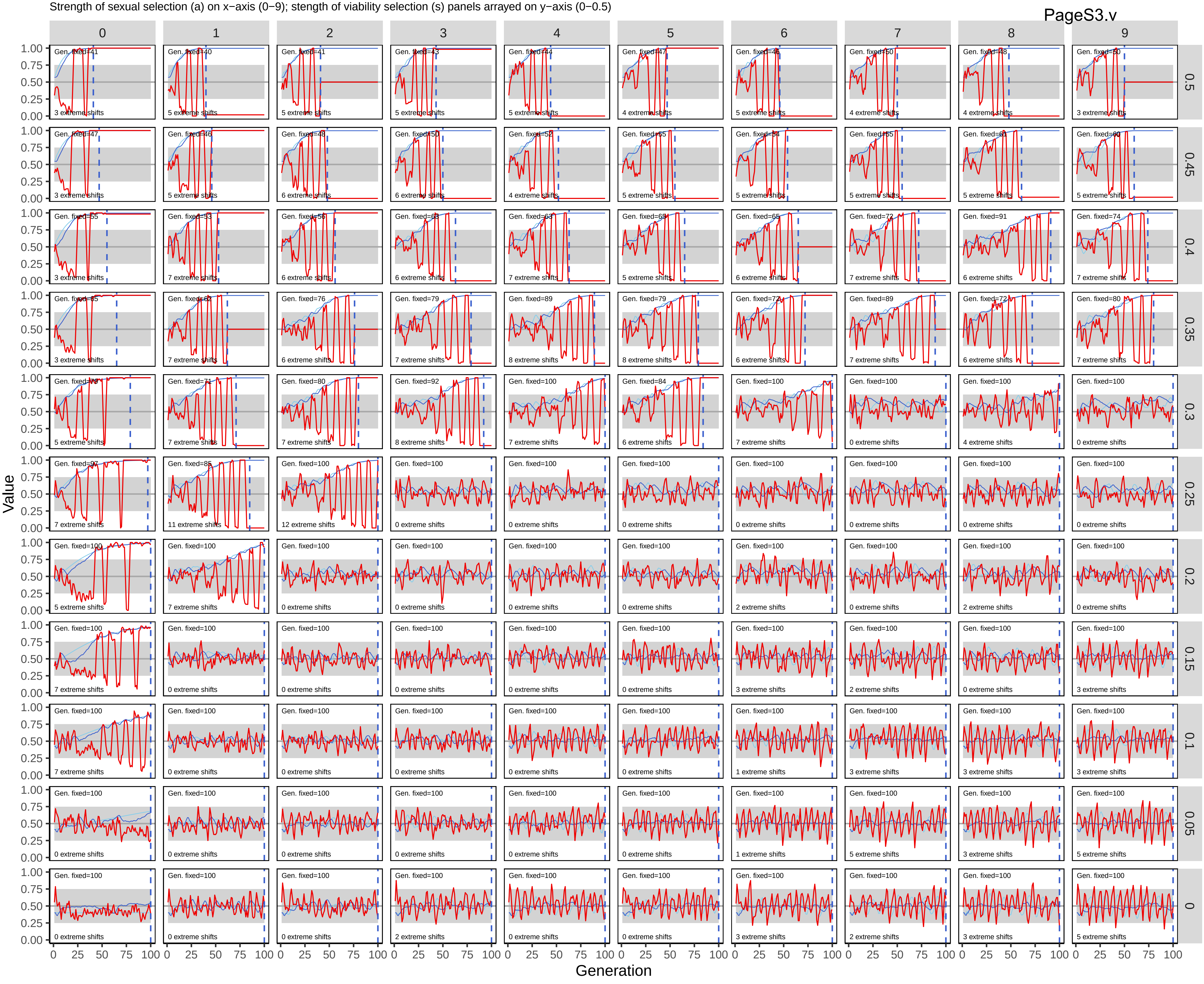

Model:fde, setting:xpt1, group size:30 (Preference=red, Trait A=dark blue, Trait B=light blue)

Strength of sexual selection (a) on x−axis (0−9); strength of viability selection (s) panels arrayed on y−axis (0−0.5)

PageS3.vi

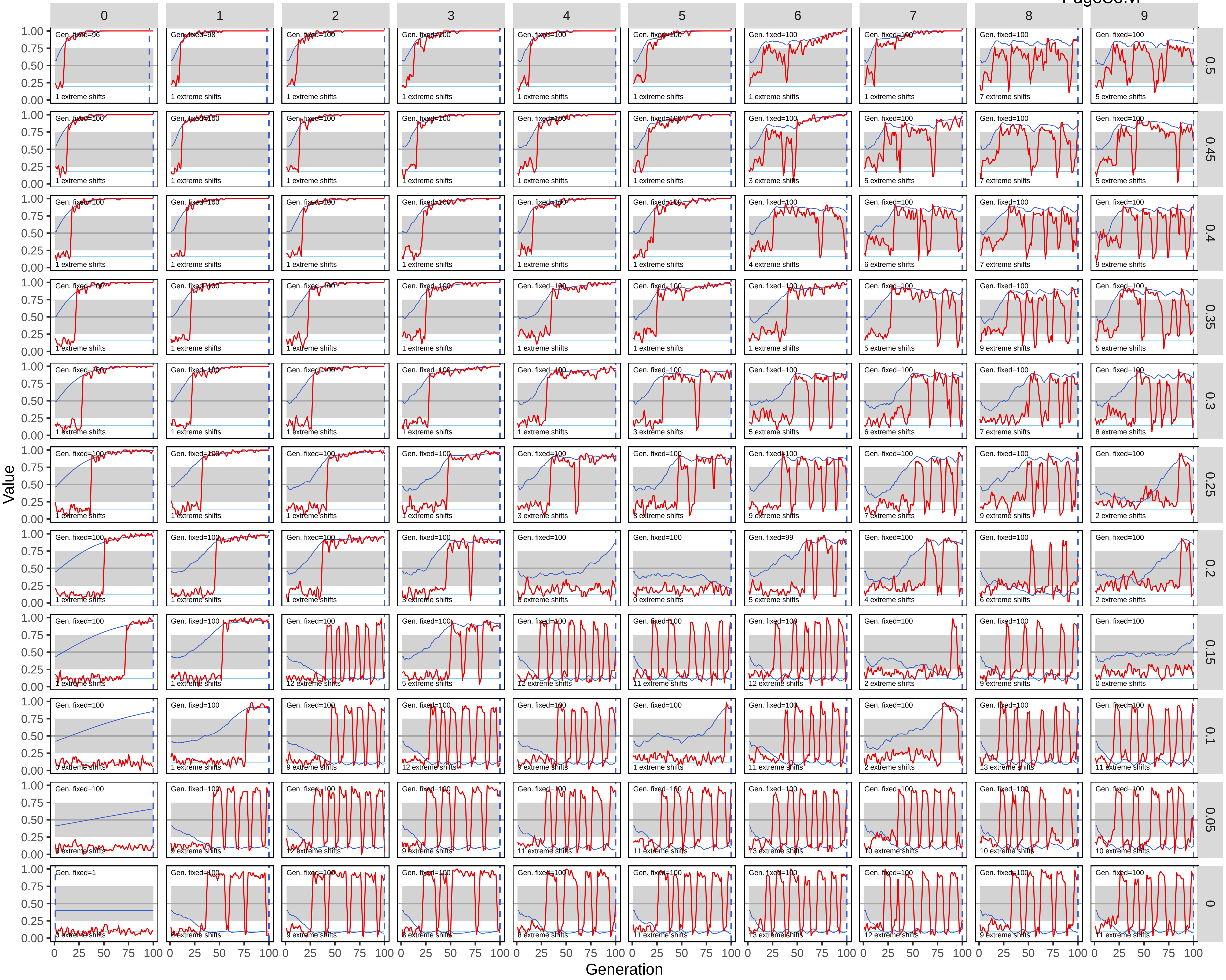

Model:fde, setting:x, group size:30 (Preference=red, Trait A=dark blue, Trait B=light blue)

Strength of sexual selection (a) on x−axis (0−9); strength of viability selection (s) panels arrayed on y−axis (0−0.5)

PageS3.vii

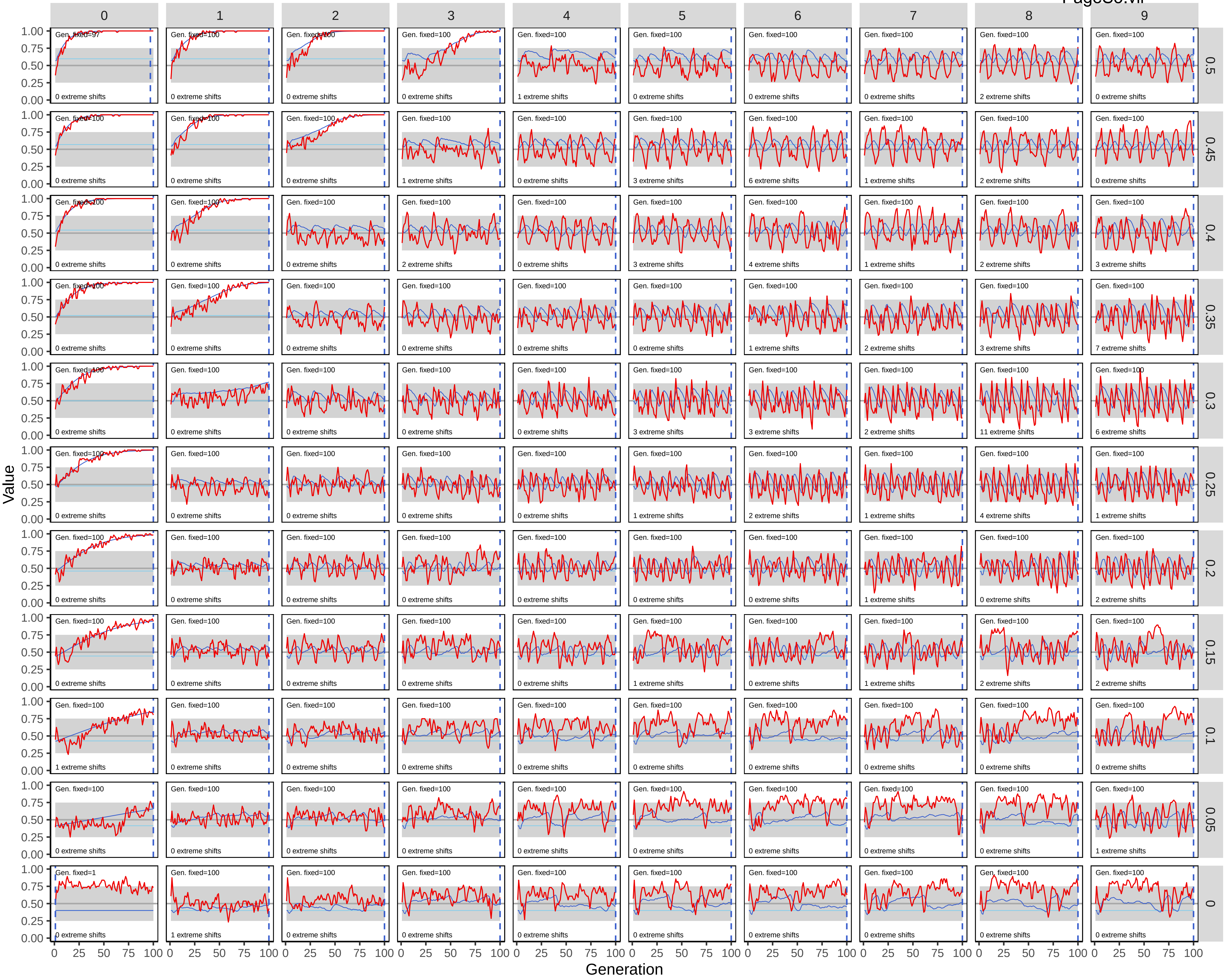

Model:sc, setting:x, group size:30 (Preference=red, Trait A=dark blue, Trait B=light blue)

Strength of sexual selection (a) on x−axis (0−9); strength of viability selection (s) panels arrayed on y−axis (0−0.5)

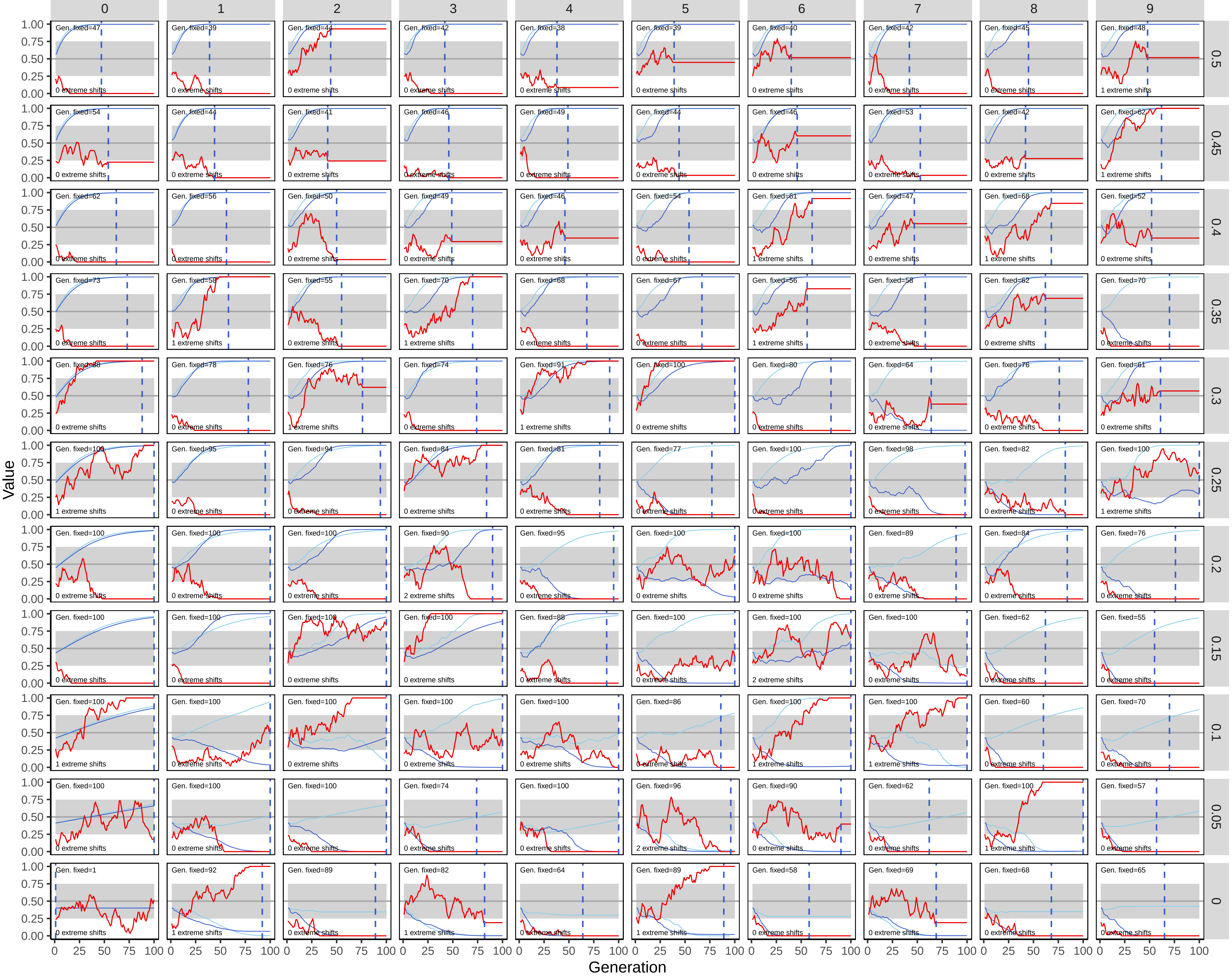

Model:ncs, setting:x, group size:30 (Preference=red, Trait A=dark blue, Trait B=light blue)

Strength of sexual selection (a) on x−axis (0−9); strength of viability selection (s) panels arrayed on y−axis (0−0.5)

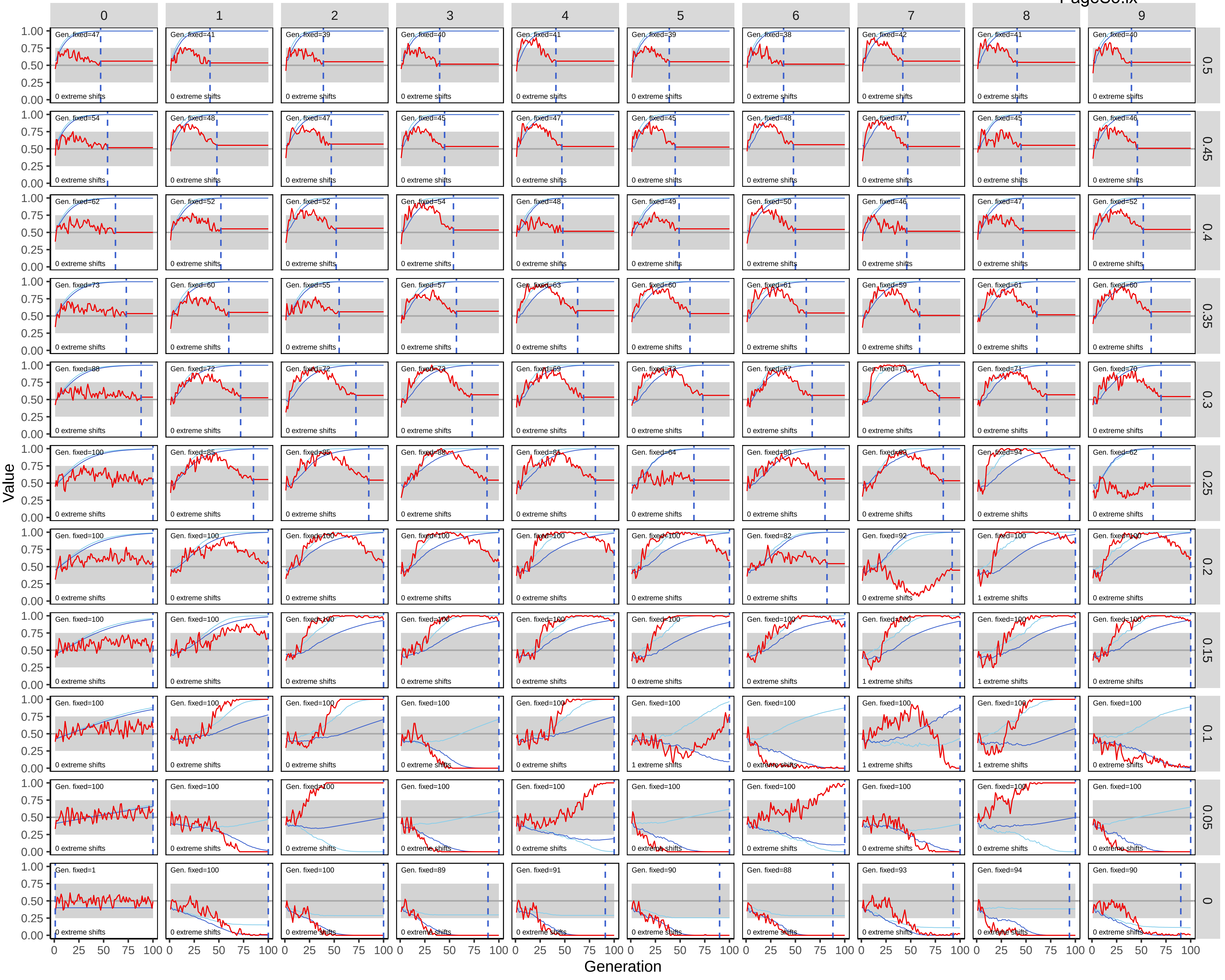

Model:pnov, setting:x, group size:30 (Preference=red, Trait A=dark blue, Trait B=light blue)

Strength of sexual selection (a) on x−axis (0−9); strength of viability selection (s) panels arrayed on y−axis (0−0.5)

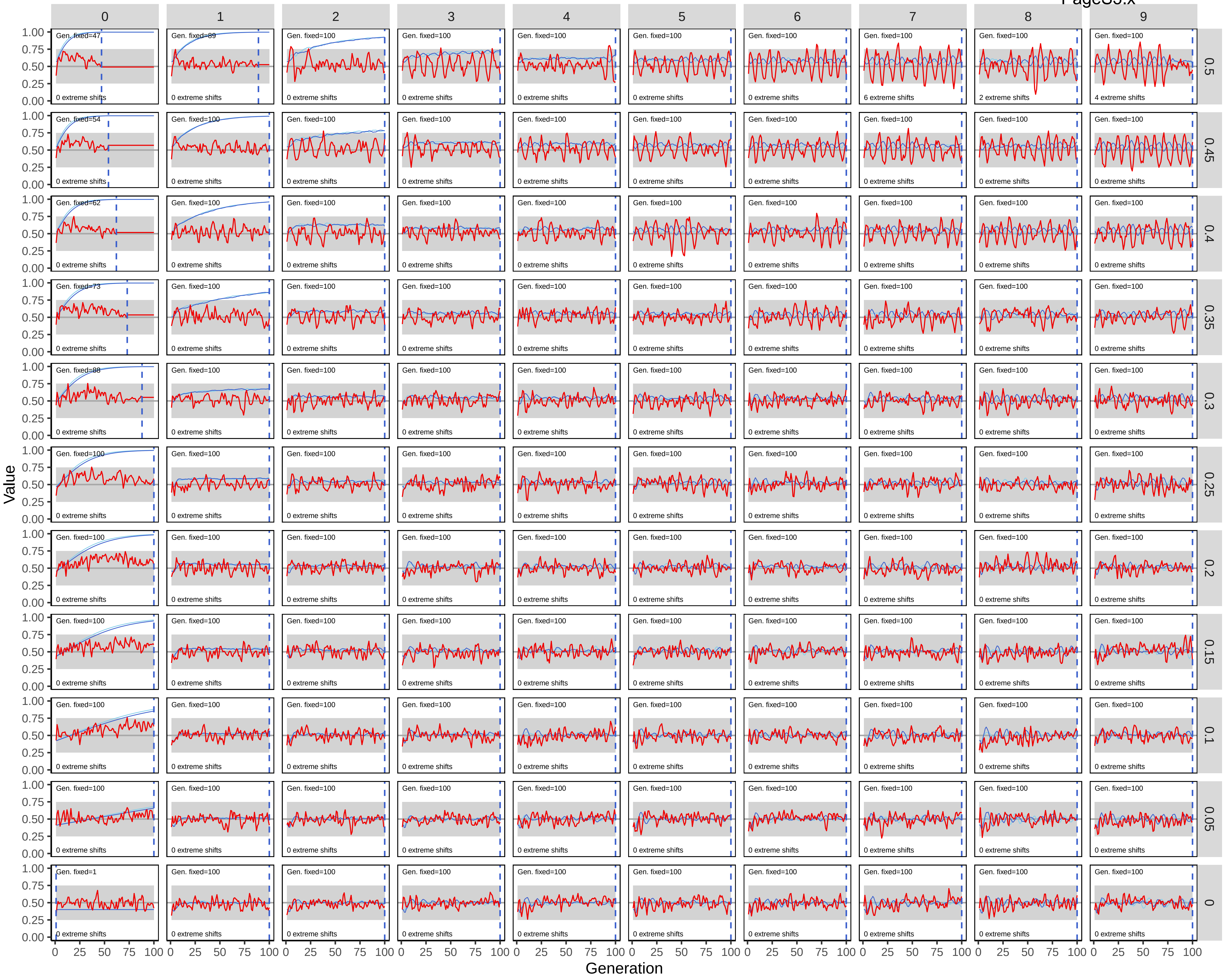

Model:pnov, setting:x, group size:100 (Preference=red, Trait A=dark blue, Trait B=light blue)

Strength of sexual selection (a) on x−axis (0−9); strength of viability selection (s) panels arrayed on y−axis (0−0.5)

PageS3.xi

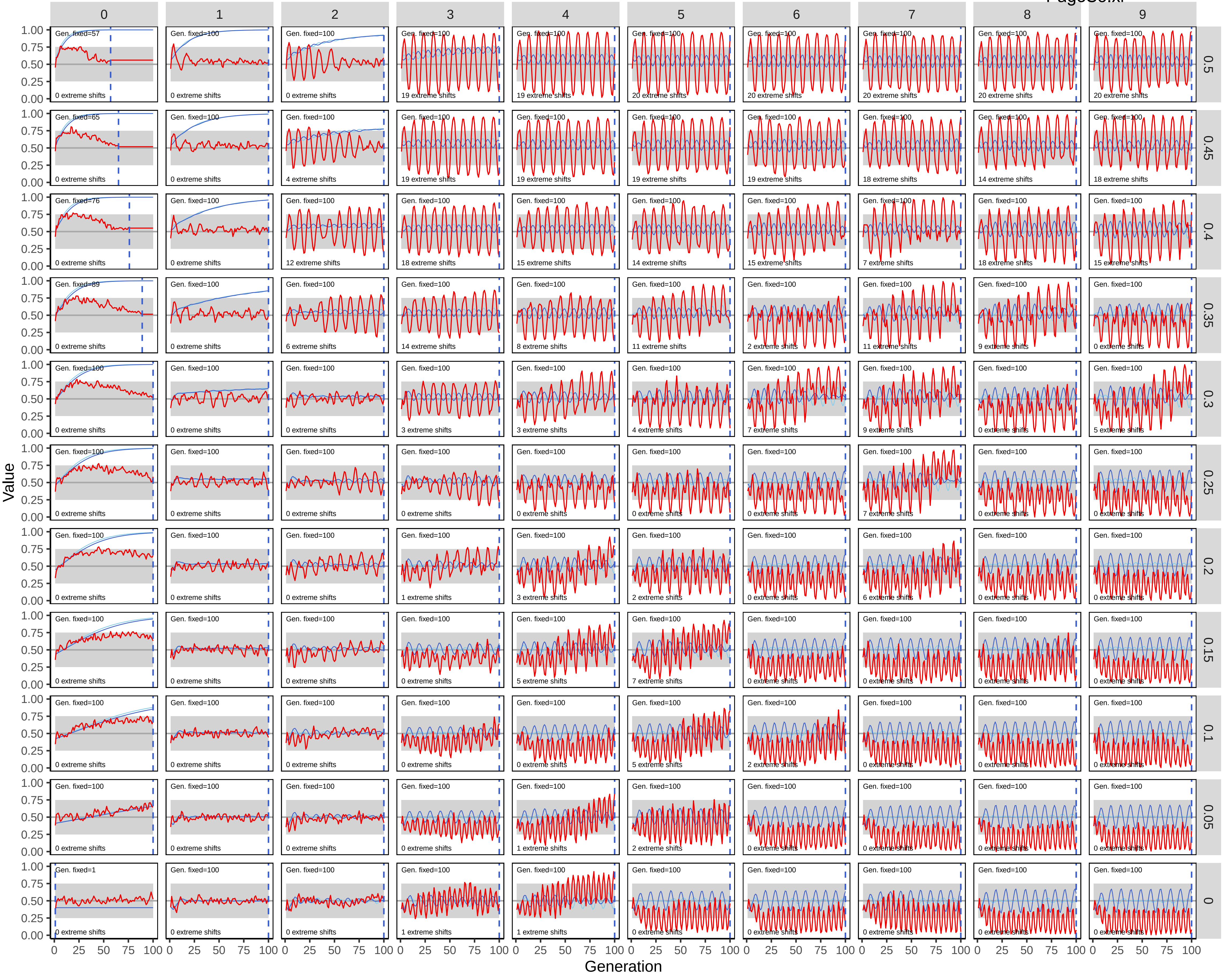

Supplement: S3 Fig — Plots show details from the full considered range of viability selection and preference strength. Output on each page represents one run of each model, and models are identified by the combination of model, setting, and group size indicated in the header on each page. Code and raw simulation output used to generate these figures are archived on the Open Science Framework; DOI: 10.17605/OSF.IO/R673J. The first page of the set of figures provides a key identifying the parameters used in each model shown. Most models are shown with male group size 30, which we considered the most biologically realistic, but we present the main model at smaller (S3.i) and larger (S3.iii) male group sizes to illustrate the effects of group size on outcomes. In general, when IA occurs in larger male group sizes, stochasticity of outcomes is reduced. We also present outcomes for model pnov.x at the larger group size (100 males, S3.xi), as this version of the model produced larger fluctuations in female preference than observed at smaller male group sizes. Grey bars along the top of each plot grid indicate the level of sexual selection (α) for subplots in that column, while grey bars along the left side of plots indicate the level of viability selection for subplots in that row. X-axes of each subplot indicate generation, and y-axes represent frequency. Red lines plot the frequency of female preference for trait TB (pB, where 1−pB females prefer trait TA); dark blue lines indicate the frequency of trait variant TA2, and light blue lines indicate frequency of TB2, with frequencies plotted after selection in each generation. Horizontal lines indicate a frequency of 0.5 for reference. Text in the upper left of each subplot indicates the last generation in which traits were variable (such that gen. fixed = 100 indicates that traits maintained variation in all plotted generations), and this generation is also indicated by a vertical dashed blue line. In S3 plots, if trait variation persisted bey [file pbio.3002269.s004.pdf]
