# Supplementary material for: Inferred Attractiveness: A generalized mechanism for sexual selection that can maintain variation in traits and preferences over time
Source: PLoS Biol. 2023 Oct 3;21(10):e3002269. doi: 10.1371/journal.pbio.3002269 (PMC10547189; doi:10.1371/journal.pbio.3002269)
Supplement: S1 Table — The mating decisions of females with the PA phenotype are influenced by male trait locus TA (here, color) and the mating decisions of females with the PB phenotype are influenced by male trait locus TB (here, pattern). The OA and OB cultural traits carry preference phenotypes for one of the alternate male alleles (1 or 2) of each type of trait (e.g., for the “striped” phenotype at the “pattern” locus), and females acquire variant information for both TA and TB from the chosen male genotype. As females attend to only one trait locus at a time, the phenotype at only one of the OA or OB positions is expressed (indicated in bold). (DOCX) [file pbio.3002269.s005.docx]

| Male Genotype | $\mathrm{TA}_{1}\mathrm{TB}_{1}$ | $\mathrm{TA}_{1}\mathrm{TB}_{2}$ | $\mathrm{TA}_{2}\mathrm{TB}_{1}$ | $\mathrm{TA}_{2}\mathrm{TB}_{2}$ |
| --- | --- | --- | --- | --- |
| Male Phenotype |  |  |  |  |
| Corresponding Female Mating Phenotype | ${\mathbf{P}_{\mathbf{A}}\mathbf{OA}}_{\mathbf{1}}\mathrm{OB}_{1}$  &  ${\mathbf{P}_{\mathbf{B}}\mathrm{OA}}_{1}\mathbf{OB}_{\boldsymbol{1}}$ | ${\mathbf{P}_{\mathbf{A}}\mathbf{OA}}_{\mathbf{1}}\mathrm{OB}_{2}$  &  ${\mathbf{P}_{\mathbf{B}}\mathrm{OA}}_{1}\mathbf{OB}_{\boldsymbol{2}}$ | ${\mathbf{P}_{\mathbf{A}}\mathbf{OA}}_{\mathbf{2}}\mathrm{OB}_{1}$  &  ${\mathbf{P}_{\mathbf{B}}\mathrm{OA}}_{2}\mathbf{OB}_{\boldsymbol{1}}$ | ${\mathbf{P}_{\mathbf{A}}\mathbf{OA}}_{\mathbf{2}}\mathrm{OB}_{2}$  &  ${\mathbf{P}_{\mathbf{B}}\mathrm{OA}}_{2}\mathbf{OB}_{\boldsymbol{2}}$ |

**S1 Table**. Model specification of male genotypes, phenotypes, and corresponding phenotypes of females that prefer these males. The mating decisions of females with the P_A_ phenotype are influenced by male trait locus TA (here, color) and the mating decisions of females with the P_B_ phenotype are influenced by male trait locus TB (here, pattern). The OA and OB cultural traits carry preference phenotypes for one of the alternate male alleles (1 or 2) of each type of trait (e.g., for the “striped” phenotype at the “pattern” locus), and females acquire variant information for both TA and TB from the chosen male genotype. As females attend to only one trait locus at a time, the phenotype at only one of the OA or OB positions is expressed (indicated in bold).
