# Supplementary material for: Inferred Attractiveness: A generalized mechanism for sexual selection that can maintain variation in traits and preferences over time
Source: PLoS Biol. 2023 Oct 3;21(10):e3002269. doi: 10.1371/journal.pbio.3002269 (PMC10547189; doi:10.1371/journal.pbio.3002269)
Supplement: S2 Table — Subscripts 1–32 are for young adult females and subscripts 33–64 are for older adult females. (DOCX) [file pbio.3002269.s006.docx]

| **Females** | **Males** | | | | | | |
| --- | --- | --- | --- | --- | --- | --- | --- |
|  |  |  | $\bar{x}_{m, 1}^{\mathrm{vs}}$ | $\bar{x}_{m, 2}^{\mathrm{vs}}$ | $\bar{x}_{m, 3}^{\mathrm{vs}}$ | $\bar{x}_{m, 4}^{\mathrm{vs}}$ |  |
|  |  |  | $\mathrm{TA}_{1}\mathrm{TB}_{1}$ | $\mathrm{TA}_{1}\mathrm{TB}_{2}$ | $\mathrm{TA}_{2}\mathrm{TB}_{1}$ | $\mathrm{TA}_{2}\mathrm{TB}_{2}$ |  |
|  | $x_{f, 1}^{\mathrm{vs}}$-$x_{f, 4}^{\mathrm{vs}}$ & $x_{f, 33}^{\mathrm{vs}}$-$x_{f, 36}^{\mathrm{vs}}$ | ${\mathbf{P}_{\mathbf{A}}\mathbf{OA}}_{\mathbf{1}}\mathbf{OB}_{\boldsymbol{1}}$ | (1+$\alpha_{a}$) | (1+$\alpha_{a}$) | 1 | 1 |  |
|  | $x_{f, 5}^{\mathrm{vs}}$-$x_{f, 8}^{\mathrm{vs}}$ & $x_{f, 37}^{\mathrm{vs}}$-$x_{f, 40}^{\mathrm{vs}}$ | ${\mathbf{P}_{\mathbf{A}}\mathbf{OA}}_{\mathbf{1}}\mathbf{OB}_{\mathbf{2}}$ | (1+$\alpha_{a}$) | (1+$\alpha_{a}$) | 1 | 1 |  |
|  | $x_{f, 9}^{\mathrm{vs}}$-$x_{f, 12}^{\mathrm{vs}}$ & $x_{f, 41}^{\mathrm{vs}}$-$x_{f, 44}^{\mathrm{vs}}$ | ${\mathbf{P}_{\mathbf{A}}\mathbf{OA}}_{\mathbf{2}}\mathbf{OB}_{\boldsymbol{1}}$ | 1 | 1 | (1+$\alpha_{a}$) | (1+$\alpha_{a}$) |  |
|  | $x_{f, 13}^{\mathrm{vs}}$-$x_{f, 16}^{\mathrm{vs}}$ & $x_{f, 45}^{\mathrm{vs}}$-$x_{f, 48}^{\mathrm{vs}}$ | ${\mathbf{P}_{\mathbf{A}}\mathbf{OA}}_{\mathbf{2}}\mathbf{OB}_{\boldsymbol{2}}$ | 1 | 1 | (1+$\alpha_{a}$) | (1+$\alpha_{a}$) |  |
|  | $x_{f, 17}^{\mathrm{vs}}$-$x_{f, 20}^{\mathrm{vs}}$ & $x_{f, 49}^{\mathrm{vs}}$-$x_{f, 52}^{\mathrm{vs}}$ | ${\mathbf{P}_{\mathbf{B}}\mathbf{OA}}_{\mathbf{1}}\mathbf{OB}_{\mathbf{1}}$ | (1+$\alpha_{b}$) | 1 | (1+$\alpha_{b}$) | 1 |  |
|  | $x_{f, 21}^{\mathrm{vs}}$-$x_{f, 24}^{\mathrm{vs}}$ & $x_{f, 53}^{\mathrm{vs}}$-$x_{f, 56}^{\mathrm{vs}}$ | ${\mathbf{P}_{\mathbf{B}}\mathbf{OA}}_{\boldsymbol{1}}\mathbf{OB}_{\boldsymbol{2}}$ | 1 | (1+$\alpha_{b}$) | 1 | (1+$\alpha_{b}$) |  |
|  | $x_{f, 25-28}^{\mathrm{vs}}$-$x_{f, 4}^{\mathrm{vs}}$ & $x_{f, 57}^{\mathrm{vs}}$-$x_{f, 60}^{\mathrm{vs}}$ | ${\mathbf{P}_{\mathbf{B}}\mathbf{OA}}_{\mathbf{2}}\mathbf{OB}_{\boldsymbol{1}}$ | (1+$\alpha_{b}$) | 1 | (1+$\alpha_{b}$) | 1 |  |
|  | $x_{f, 29-32}^{\mathrm{vs}}$-$x_{f, 4}^{\mathrm{vs}}$ & $x_{f, 61}^{\mathrm{vs}}$-$x_{f, 64}^{\mathrm{vs}}$ | ${\mathbf{P}_{\mathbf{B}}\mathbf{OA}}_{\mathbf{2}}\mathbf{OB}_{\mathbf{2}}$ | 1 | (1+$\alpha_{b}$) | 1 | (1+$\alpha_{b}$) |  |

S2 Table. Coefficients determining non-random mating (the numerators in equations 2 and 3) across all combinations of mated genotype pairs in the Inferred Attractiveness model. Subscripts 1-32 are for young adult females and subscripts 33-64 are for older adult females.
