# Supplementary material for: Inferred Attractiveness: A generalized mechanism for sexual selection that can maintain variation in traits and preferences over time
Source: PLoS Biol. 2023 Oct 3;21(10):e3002269. doi: 10.1371/journal.pbio.3002269 (PMC10547189; doi:10.1371/journal.pbio.3002269)
Supplement: Attachment — Submitted filename: DuVal et al - RESPONSE - FINAL 15Jul23.docx [file pbio.3002269.s007.docx]

-------------------------------------------------------------------------------------------------------------------------------

RESPONSE TO EDITORIAL AND REVIEWER COMMENTS

Our line-by-line responses are preceded with “Author response:” Line numbers listed here refer to the tracked changes version of the final manuscript

-------------------------------------------------------------------------------------------------------------------------------

Dear Dr DuVal,

Thank you for your patience while your manuscript "Inferred Attractiveness and the evolutionary dynamics of mate choice in a social context" was peer-reviewed at PLOS Biology. It has now been evaluated by the PLOS Biology editors, an Academic Editor with relevant expertise, and by three independent reviewers.

Based on the reviews, which are overwhelmingly positive, we are likely to accept this manuscript for publication, provided you satisfactorily address the remaining points raised by the reviewers and the following data and other policy-related requests.

IMPORTANT:

Please attend to the following:

a) Please could you change the Title to something more accessible and appealing to a broader readership? We suggest something like "Inferred attractiveness: females may acquire mating preferences by observing others’ choices in a social context"

**Author response:** We appreciate this feedback, and we discussed an alternate title at length. How females acquire preferences is a starting point of our paper, but the part of this work that we feel is truly revolutionary is what happens when that mechanism is applied over time, which is not captured by the suggested alternative. We therefore suggest the new title: “Inferred Attractiveness: a generalized mechanism for sexual selection that can maintain variation in traits and preferences over time.” We hope that our new title is both accessible and clearly reflects this part of the work.

b) Please attend to concerns raised by the three reviewers.

**Author response:** Point-by-point responses to reviewer concerns and suggestions are below.

c) Please address my Data Policy requests below; specifically, we need you to supply the numerical values underlying Figs 1BCD, 2ABCD, 3AB, 4ABC, S1ABC, S2ABCD, S3i-xi, either as a supplementary data file or as a permanent DOI’d deposition. I note that you intend to deposit the Mathematica code in Dryad. Please could you clarify whether this will be sufficient to reproduce your Figures? If not, please supply additional data required. Please ensure that we have access to the Dryad deposition so that we can check it before accepting the manuscript for publication.

**Author response:** We have added files containing the numerical values for figures Figs 1BCD, 2ABCD, 3AB, 4ABC, S1ABC, S2ABCD, S3i-xi, as requested, as a DOI’d deposition in the Open Science Framework (OSF; DOI 10.17605/OSF.IO/R673J), and have included the Mathematica code of our models as well as the code used to compile figures from the data produced by 20 runs of each plotted simulation. We also uploaded the code and data to a GitHub repository to facilitate running the code, and will link that to our OSF files when the GitHub repository is made public. These are sufficient to reproduce our figures.

d) Please cite the location of the data clearly in all relevant main and supplementary Figure legends, e.g. “The data underlying this Figure can be found in S1 Data” or “The data underlying this Figure can be found in [https://urldefense.com/v3/__https://doi.org/10.5061/dryad.XXXXX__;!!PhOWcWs!1_C9QumoKrkkYS22F-v0qeUglSxuFsZPdmNKEdNexHk5B9nuTuL11hVz3PlUoXrkK9SyTvAWdWlLOELOfAw$](https://urldefense.com/v3/__https:/doi.org/10.5061/dryad.XXXXX__;!!PhOWcWs!1_C9QumoKrkkYS22F-v0qeUglSxuFsZPdmNKEdNexHk5B9nuTuL11hVz3PlUoXrkK9SyTvAWdWlLOELOfAw$) ”

**Author response:** This information has been added to the figure legends of Figs 1BCD, 2ABCD, 3AB, 4ABC, S1ABC, S2ABCD, S3i-xi, as requested. We also added a “script guide” to both OSF and the GitHub repository to clarify which scripts produce which figures from which datasets.

As you address these items, please take this last chance to review your reference list to ensure that it is complete and correct. If you have cited papers that have been retracted, please include the rationale for doing so in the manuscript text, or remove these references and replace them with relevant current references. Any changes to the reference list should be mentioned in the cover letter that accompanies your revised manuscript.

**Author response:** We cross-checked our references by searching each title against the retractiondatabase.org and hereby verify that none of the cited papers have been retracted. References were generated using Endnote, and all cited papers are listed in the references cited.

We expect to receive your revised manuscript within two weeks.

To submit your revision, please go to [https://urldefense.com/v3/__https://www.editorialmanager.com/pbiology/__;!!PhOWcWs!1_C9QumoKrkkYS22F-v0qeUglSxuFsZPdmNKEdNexHk5B9nuTuL11hVz3PlUoXrkK9SyTvAWdWlL2z5WYBc$](https://urldefense.com/v3/__https:/www.editorialmanager.com/pbiology/__;!!PhOWcWs!1_C9QumoKrkkYS22F-v0qeUglSxuFsZPdmNKEdNexHk5B9nuTuL11hVz3PlUoXrkK9SyTvAWdWlL2z5WYBc$)  and log in as an Author. Click the link labelled 'Submissions Needing Revision' to find your submission record. Your revised submission must include the following:
-  a cover letter that should detail your responses to any editorial requests, if applicable, and whether changes have been made to the reference list
-  a Response to Reviewers file that provides a detailed response to the reviewers' comments (if applicable)
-  a track-changes file indicating any changes that you have made to the manuscript.

NOTE: If Supporting Information files are included with your article, note that these are not copyedited and will be published as they are submitted. Please ensure that these files are legible and of high quality (at least 300 dpi) in an easily accessible file format. For this reason, please be aware that any references listed in an SI file will not be indexed. For more information, see our Supporting Information guidelines:
[https://urldefense.com/v3/__https://journals.plos.org/plosbiology/s/supporting-information__;!!PhOWcWs!1_C9QumoKrkkYS22F-v0qeUglSxuFsZPdmNKEdNexHk5B9nuTuL11hVz3PlUoXrkK9SyTvAWdWlL0wMmL0U$](https://urldefense.com/v3/__https:/journals.plos.org/plosbiology/s/supporting-information__;!!PhOWcWs!1_C9QumoKrkkYS22F-v0qeUglSxuFsZPdmNKEdNexHk5B9nuTuL11hVz3PlUoXrkK9SyTvAWdWlL0wMmL0U$)   

*Published Peer Review History*
Please note that you may have the opportunity to make the peer review history publicly available. The record will include editor decision letters (with reviews) and your responses to reviewer comments. If eligible, we will contact you to opt in or out. Please see here for more details:
<https://urldefense.com/v3/__https://blogs.plos.org/plos/2019/05/plos-journals-now-open-for-published-peer-review/__;!!PhOWcWs!1_C9QumoKrkkYS22F-v0qeUglSxuFsZPdmNKEdNexHk5B9nuTuL11hVz3PlUoXrkK9SyTvAWdWlLMdOU6Ac$>

**Author response:** We would indeed like to opt in to public peer review history, and will watch for this opportunity.

*Press*
Should you, your institution's press office or the journal office choose to press release your paper, please ensure you have opted out of Early Article Posting on the submission form. We ask that you notify us as soon as possible if you or your institution is planning to press release the article.

**Author response:** We have opted out of early article posting, and will be working with FSU’s public communications department on a press release once a final acceptance decision is received. We will notify you when this press release is drafted and its release is planned.

*Protocols deposition*
To enhance the reproducibility of your results, we recommend that if applicable you deposit your laboratory protocols in [protocols.io](http://protocols.io/), where a protocol can be assigned its own identifier (DOI) such that it can be cited independently in the future. Additionally, PLOS ONE offers an option for publishing peer-reviewed Lab Protocol articles, which describe protocols hosted on [protocols.io](http://protocols.io/). Read more information on sharing protocols at [https://urldefense.com/v3/__https://plos.org/protocols?utm_medium=editorial-email&utm_source=authorletters&utm_campaign=protocols__;!!PhOWcWs!1_C9QumoKrkkYS22F-v0qeUglSxuFsZPdmNKEdNexHk5B9nuTuL11hVz3PlUoXrkK9SyTvAWdWlLjoknCLg$](https://urldefense.com/v3/__https:/plos.org/protocols?utm_medium=editorial-email&utm_source=authorletters&utm_campaign=protocols__;!!PhOWcWs!1_C9QumoKrkkYS22F-v0qeUglSxuFsZPdmNKEdNexHk5B9nuTuL11hVz3PlUoXrkK9SyTvAWdWlLjoknCLg$)

**Author response:** not applicable for this article.

Please do not hesitate to contact me should you have any questions.

Sincerely,

Roli Roberts


Roland Roberts, PhD
Senior Editor,
[rroberts@plos.org](mailto:rroberts@plos.org),
PLOS Biology

------------------------------------------------------------------------
DATA POLICY:

You may be aware of the PLOS Data Policy, which requires that all data be made available without restriction: [https://urldefense.com/v3/__http://journals.plos.org/plosbiology/s/data-availability__;!!PhOWcWs!1_C9QumoKrkkYS22F-v0qeUglSxuFsZPdmNKEdNexHk5B9nuTuL11hVz3PlUoXrkK9SyTvAWdWlLZMyE2I8$](https://urldefense.com/v3/__http:/journals.plos.org/plosbiology/s/data-availability__;!!PhOWcWs!1_C9QumoKrkkYS22F-v0qeUglSxuFsZPdmNKEdNexHk5B9nuTuL11hVz3PlUoXrkK9SyTvAWdWlLZMyE2I8$) . For more information, please also see this editorial: [https://urldefense.com/v3/__http://dx.doi.org/10.1371/journal.pbio.1001797__;!!PhOWcWs!1_C9QumoKrkkYS22F-v0qeUglSxuFsZPdmNKEdNexHk5B9nuTuL11hVz3PlUoXrkK9SyTvAWdWlL0CO3ffc$](https://urldefense.com/v3/__http:/dx.doi.org/10.1371/journal.pbio.1001797__;!!PhOWcWs!1_C9QumoKrkkYS22F-v0qeUglSxuFsZPdmNKEdNexHk5B9nuTuL11hVz3PlUoXrkK9SyTvAWdWlL0CO3ffc$) 

Note that we do not require all raw data. Rather, we ask that all individual quantitative observations that underlie the data summarized in the figures and results of your paper be made available in one of the following forms:

1) Supplementary files (e.g., excel). Please ensure that all data files are uploaded as 'Supporting Information' and are invariably referred to (in the manuscript, figure legends, and the Description field when uploading your files) using the following format verbatim: S1 Data, S2 Data, etc. Multiple panels of a single or even several figures can be included as multiple sheets in one excel file that is saved using exactly the following convention: S1_Data.xlsx (using an underscore).

2) Deposition in a publicly available repository. Please also provide the accession code or a reviewer link so that we may view your data before publication.

Regardless of the method selected, please ensure that you provide the individual numerical values that underlie the summary data displayed in the following figure panels as they are essential for readers to assess your analysis and to reproduce it: Figs 1BCD, 2ABCD, 3AB, 4ABC, S1ABC, S2ABCD, S3i-xi. NOTE: the numerical data provided should include all replicates AND the way in which the plotted mean and errors were derived (it should not present only the mean/average values).

IMPORTANT: Please also ensure that figure legends in your manuscript include information on where the underlying data can be found, and ensure your supplemental data file/s has a legend.

Please ensure that your Data Statement in the submission system accurately describes where your data can be found.

**Author response:** addressed above; we have also updated the Data Statement in the submission system to reflect that our data and code repository is now on OSF, not Dryad.

------------------------------------------------------------------------
DATA NOT SHOWN?

- Please note that per journal policy, we do not allow the mention of "data not shown", "personal communication", "manuscript in preparation" or other references to data that is not publicly available or contained within this manuscript. Please either remove mention of these data or provide figures presenting the results and the data underlying the figure(s).

**Author response:** not applicable for this article – our manuscript does not include "data not shown", "personal communication", "manuscript in preparation" or other references to data that is not publicly available or contained within this manuscript

------------------------------------------------------------------------

REVIEWERS' COMMENTS:


Reviewer #1:

I reviewed manuscript PBIOLOGY-D-23-01008R1, "Inferred Attractiveness and the evolutionary dynamics of mate choice in a social context." The authors argue that, when individuals learn mate preferences by observing the mate choices of others, they may learn preferences for the most distinguishing traits of the chosen mates rather than for the traits that the original choosers used in their mate assessment. Using a mathematical model, the authors show that this can set up patterns of fluctuating and mostly negative frequency dependent mate preference at the population level, and that this can maintain multiple mate phenotypes in the population, even when those phenotypes are disfavored by viability selection. Most previous models predict that sexual selection due to learned or genetic mate preferences will squeeze variability out of mating traits, and it has been a long-standing paradox that variability in mating traits is common in nature. This paper offers a solution to that paradox. The authors' model is simple and compelling. The work falls into the class of results that we probably should have known but apparently didn't, which is the sweet spot for new theory. Moreover, the manuscript itself is clear and well-written. I believe this paper will change the way we think about sexual selection and set off a flurry of new theoretical and empirical work in this and related areas.

**Author response:** We greatly appreciate this positive assessment of our work.

I have made a few minor suggestions below, but I want to be clear that these are just suggestions. I hope the authors can find something useful in them, but they are (as always!) free to take or leave them.

Many of my suggestions stem from the observation that the methods section of this paper presents a lot of detail on how the model is coded. This may be useful for people who think in code, but may make the paper less accessible to theory savvy biologists who are not proficient programmers. For example, the authors tell us in where various pieces of information are stored in various vectors and matrices, but do we really need to know that? Would this not be exactly the same model if the authors had computed the frequencies of mating pairs using nested "for" loops rather than matrix operations? If so, then I think the authors might be better to present just the information that readers would need to understand the mathematical model in the methods, and to save the details of how they implemented that model for the annotated Mathematica notebook. I recognize that this is a matter of personal taste, and the authors should not have to do it or even respond to it. But, I encourage them to spend a few minutes thinking about what the paper might look like if the methods explained just the model, with less detail on how the model was implemented in their code.

**Author response:** We appreciate this suggestion, and the general focus here on improving accessibility of the paper. We discussed this at length, and read through the paper revising details throughout to reduce the emphasis on the programming details of the model (e.g., line 484-506), but do feel that it is nevertheless essential to provide this information. There are thus many changes throughout the model exposition; other examples can be seen in paragraphs 2-5 of the methods. However, if the editor allows, we would prefer not to further change the presentation of model coding details that are currently part of the main paper.

We note that while some of the details of the model were written to capture the programming implementation (for example, the storage of both age classes in a single vector) other details such as the use of vectors or matrices at all are extremely standard in population genetic model exposition when it is done by genotype instead of allele frequency, and would have to remain even if we changed the style of the write-up substantially. We note that we also provide verbal descriptions of all aspects of the model in terms of alleles/cultural phenotypes (including below each equation; see also, e.g., Table S2), so we hope that we are striking a balance in this way.

Minor comments

Lines 143-144: The authors tell us that variability in the male traits in their model can be "lost" due to viability selection. This made me wonder about what causes fixation of genetic loci in this model. Does fixation occur stochastically despite negative frequency-dependent selection (eg, in generations when no females learn to prefer the rare male phenotype)? Or, is there some basin of attraction around a set of polymorphic states, and if the system wanders out of that basin does it proceed (in the absence of further stochasticity) deterministically to fixation of the male traits? I suspect one could answer this by studying the rates of change at the male trait loci when two of the male phenoptypes are vanishingly rare. I don't think the authors should spend days and pages figuring this out - if it is that much work it is probably a new project - but if they can answer it quickly and easily then it might help readers to understand the behavior of the system.

**Author response:** Thanks for the question, this has helped us to clarify this point in the text. While we cannot analyze the model analytically, we can speculate on these points based on the examination of replicate simulation results. There is an equilibrium at a polymorphic state (though this may be a limit cycle rather than a point, judging from visual evidence) and an equilibrium at fixation of the allele favored by natural selection. In some cases, the former equilibrium either does not exist or is unstable, namely when viability selection is a sufficient amount stronger than sexual selection. However, even when the polymorphic equilibrium is stable, the system can, as the reviewer speculated, wander outside of its basin of attraction to the basin of attraction at fixation. Therefore there are some parameter values (such as s = 0.4, a = 6) where variation is maintained in some replicates but lost in others. We have elaborated on this very briefly in line 183 and in more depth in the new short paragraph on lines 228-236.

Figure 2: Does this figure illustrate the exact same simulations as Figure 1b? If so, that could be stated clearly.

**Author response:** Figure 2 shows a different replicate of the same model shown in figure 1B. We have added text to the figure legend to clarify this. As the output of each model is stochastic, numbers are not precisely the same.

Figure 3: In 3ai, do the bottom two panels show the same conditions (but different replicates) as the bottom two panels in 1b? And, in 3bi, do the left two panels show the same conditions (but different replicates) as the left two panels in 1b? If so, I would recommend either stating that clearly or removing the panels (since the goal of the figure is to show what happens when selection on the traits differs, and in these panels I think it does not differ).

**Author response:** Yes, this is the case. We have added text to the legend of figure 3 to make this clear (lines 282-285).

Also, should the headings of the right columns in 3ai and 3bi be "alpha = 7?"

**Author response:** Thank you for catching this mistake. We have now fixed this header.

I guess there were 20 replicates for each combination of parameter values, but it would help readers if this were stated in the caption or legend.

**Author response:** We have added text to the legend of figure 3 to indicate that this is the case (lines 279-282).

Figure 4: When one trait is environmentally determined and viability selection on the genetic trait is not strong enough to overcome sexual selection, it makes intuitive sense to me that the frequency of the genetic trait gets "pinned" to the frequency of the environmentally determined trait. But, is there anything special about which allele gets pinned at low density? In the top right of 4a (ie, alpha=7, s=0.4), the favored allele is more common, which makes sense because viability selection is reasonably strong. In the bottom panels, where viability selection is weak, the disfavored allele is more common. But, does it have to be that way, or does it vary among replicates? These details might help readers more fully understand how the system behaves.

**Author response:** The reviewer is right that it can vary among replicates. In some cases, the favored allele is more common when viability selection is weak, for example (although this is relatively rare). We have now made this clear in the legend to Figure 4.

Line 352: Referring to the behavioral phenotypes as "loci" is confusing for me. I know the phenotypes have been coded like loci, but biologically they are not loci. I think this may be especially confusing for readers who are biologists and perhaps do not do a lot of coding - which is likely to be most of them, since I think this paper will have a broad readership. If I understand the model correctly, all of the important behavioral information about females can be captured by specifying the male phenotype they prefer. So, if it were my paper, I would describe females by just their genotype, their mate preference (as a single phenotype rather than three), and their age (even if, for computational convenience, I had tracked these things in a more complicated way).

**Author response:** We thank the reviewer for this suggestion. We have changed the reference to behavioral phenotypes from “loci” and “alleles” to refer to them respectively as “cultural traits” and “cultural trait variants”. The shorthand of “trait” and “trait-variant” can therefore be used for these cultural traits in the same way that they are in the main text for the genetic traits TA and TB and the genetic trait variants TA_1_, TA_2_, TB_1_ and TB_2_.

We agree that it would be nice if we could describe the mate preference as a single phenotype, but this would still need two axes of information, which trait is focused upon (TA or TB) and which trait variant (at that trait of interest, e.g. TA_1_ vs TA_2_ at locus TA) is preferred. Since we plot both of these in different places (e.g., the former as the red lines on Figures 1, 3, and 4 and the latter in Figure 2), and both of these pieces of information are necessary for understanding the changes in preference phenotypes (e.g., Figure 2), we have to keep them separate. We have therefore opted to stick with the current scheme, although we are now clearer about there only being two relevant axes of information in lines 446-447.

Line 433: If I understand correctly, wbar here is not mean fitness, it is just mean survivorship between age classes. Fitness would include reproductive success. If that is true, then I recommend calling this mean survivorship and replacing w with some other variable, because many readers will expect w to be fitness. I also suspect that the model could be presented more simply if the authors did not try to index as in their code, but instead used, for example, m_ij(t) to indicate males born in generation t with alleles i and j at the TA and TB loci. Then I think they could define z_ij as the survivorship between age classes for males with genotype ij, and the proportion of males with genotype ij in the mating pool in generation t would just be m_ij(t-1) z_ij + m_ij(t-2) z_ij^2 divided by sum(m_xy(t-1) z_xy + m_xy(t-2) z_xy^2) for x and y in (1,2). Then, I do not think the authors would need equation 1 at all. I have not tried to rewrite the whole methods section using this notation, so maybe starting out this way would lead to trouble later, but if so I don't see it.

**Author response**: The reviewer is correct that the wbar used in the text is mean survivorship, and does not include reproductive success. We have therefore changed the notation to indicate that this is mean fitness through viability selection alone by adding a “v” as a superscript, and have called it “mean survivorship” instead of “mean fitness” (see lines 551-552).

We understand that the reviewer has a preference for different notation for the model presentation, but we have opted to stick with the current notation since it is very common in population genetic models, and we worry that changing the notation with such a short time frame for revisions would make us liable to the introduction of errors. The reviewer’s formulation of equation 2 above is also not equivalent to our version of equation 2 (checked numerically).

Line 574: "…while preserving any linkage disequilibrium that has built up between TA and TB." But, here, TB is an environmentally determined trait. So, what does a linkage disequilibrium represent in this case? Is there some assumption being made about offspring sharing the parental environment, and therefore the parental environmental trait? If not, I would think that no linkage disequilibrium could arise. In any case, it would help me as a reader to have the biological motivation for maintaining this linkage disequilibrium more clearly explained.

**Author response**: Thanks to the reviewer for this comment. This was an error – the LD preserved in the code was between TA and C, a locus which was included in the code to allow additional assumptions to be made to the model, but which was never “turned on” in the analyses that we ran for this paper (which had already become quite complex as a “baseline” model. We have removed this phrase from the text.

Reviewer #2:
[identifies himself as Gil G. Rosenthal]

This is an inspired and exciting model that breaks fundamental new ground in the way theoreticians have approached sexual selection. The model explicitly considers the complex interaction between public and private information, and shows that "inferred attractiveness" can explain much of the dynamism and variability observed in mate preferences in nature.  This is a refreshing break from tweaks on the "good genes vs runaway" wrestling match that has dominated sexual selection theory. A pioneering model like this can only do so much in terms of exploring assumptions and parameter space. While the comparison to reference models is thorough and important, I think a little more discussion about the specificity and generality of the key assumptions would be helpful.  To be clear, I think this is a rich model that goes beyond the unaided intuition to generate testable predictions, and I am not advocating more modeling....just perhaps a more nuanced approach to discussing the way the model does (and doesn't) map on to biology. I go through each of these in detail in the minor comments below.

1. It is refreshing that the preferences here do not have a simple genetic basis like in so many models. But to say that "genetically inherited preferences...lack widespread support" is misleading, particularly beyond vertebrates.  A broad reading of the literature suggests that G X E effects are ubiquitous in shaping mating preferences much as they do other complex traits. Further, in many systems preferences are learned through early experience with relatives, so that there is a strong indirect genetic effect of parental phenotype on mating preferences; Servedio's work with Verzijden and others shows that this kind of vertically-transmitted preference is a more powerful driver of trait-preference coevolution than genetically specified traits and preferences.  I understand that preferences aren't evolving in this model for the sake of simplicity, but part of "casting the female brain as the central selective agent" should be an acknowledgment that brains are evolving phenotypes like everything else.

**Author response**: We do agree that brain structure and function, and the responses to stimuli that they organize are at their heart genetic, and we have attempted to strike a balance between acknowledging this yet pointing out the unrealistic simplicity assumed in most models of mate choice that -- either for the sake of simplicity or because no one ever considered an alternative -- include genetic preferences as a starting assumption. To avoid overstepping, we have reworded the abstract (lines 25-26) to emphasize that we are objecting to assuming a solely genetic basis to female preferences. We attempted to address this point directly in the previous draft of the “implications and conclusions” section of the paper, but given this comment and those below, we see that we were not sufficiently clear. We have revised this final section of the paper to explain more clearly that our “non-genetic” mechanism does of course have a genetic underpinning, as it is mediated by brain function (Lines 406-413).

2. Line 64. The paradox of the lek is not so much that there isn't any trait variation, but that the consequences of mating with one courter are no different from mating with another courter, and therefore there's no incentive to invest in mate choice.

**Author response**: Historically, publications on “the paradox of the lek”, have used two interrelated formulations. One formulation focused on the predicted loss of male trait variation when there are directional preferences (and yet trait variation paradoxically persists) – e.g. the focus of Rowe and Houle’s work on condition-dependent male traits as a solution to the lek paradox. Alternately, this paradox is sometimes focused on the prediction that when trait variation is lost, it should not matter which males females choose (and yet they remain paradoxically choosy). Some studies have productively focused on this part of the paradox, e.g. Vitousek et al. 2007 doi:10.1371/journal.pone.0000567 . The reviewer is focusing here on the latter part of the paradox, but we would like to acknowledge both versions, as we feel they complement each other yet are often considered as separate components. However, to avoid any misunderstanding we have combined them into one general statement about the Paradox of the Lek (lines 63-69).

3. Line 77. The "rare male" effect - mate choice favoring rare phenotypes - has a long history in the literature (see e.g. O'Donald 1977 Nature, and Potter et al Science just this year). A little more thorough discussion of this phenomenon would be helpful, including an acknowledgement that choosers often prefer familiar phenotypes. See for example Alonzo and Sinervo, BES 2001 for an example where the fitness consequences of choosing rare males are variable for females.  It's also worth mentioning that preferences for novel "bling" are generally anchored by stabilizing preferences for traits that are species-typical or that are predictive of sexual maturity or physical health (all of which can be explained by direct selection on female fitness rather than "good genes").  And if choosers prefer novel traits within this envelope, then one would expect rare mutations to be heavily favored. In the model, of course, there are only four possible trait combinations. How would a steady influx of novel trait variants change the model?

**Author response**: We have added more text about rare male effects in line L395-397. We address the rare male effect in our comparison with a model for novelty preference, but this comment drew our attention to the fact that we actually modeled rarity, not novelty. We have added a note to clarify this in line 301, and added specific mention of preference for rare males as an indicator that females attend to distinctive traits in lines 148-151. If there were a steady influx of novel traits, female preferences would leapfrog from trait to new trait; and if males varied in many different traits, female preference And if males simply had many variable traits (which seems more plausible in a real animal) we expect low consensus in female mating preferences and sequential sexual selection on multiple traits over time, with the result that males should have elaborated traits that are not necessarily the current targets of female choice. This is in the pre-existing text on lines 403-405.

4. Figure 1b please use a third color rather than a different shade of blue for the second male trait.

**Author response**: We discussed this suggestion but would prefer to represent traits as dark and light blue rather than introducing a third color. We feel this is reasonable because both the dark and light blue lines represent traits; because the current differences reproduce well if printed in B&W; and this change would be iterated across several hundred plots (given its inclusion in all heatmap plots and the extensive supplemental figure of exemplar plots at all model conditions). Without a firm reason for why a different color is necessary, we would prefer not to change this plotting choice.

5. line 328 choosers in nature exhibit variation not only in the strength but also the direction in which social experience affects preferences. Consider satin bowerbirds, where a high energy courtship display is attractive to older females and aversive to younger females, or the sister highland and sheepshead swordtails, which reject and favor familiar phenotypes, respectively.

**Author response**: This is true, but is a somewhat different point than is being made in line 328. The noted passage highlights the probable existence of variation in strength of social learning that is not included in our model, but which represents a promising area for further development of the ideas here. If some females learn more intensely from others, do their genotypes increase in frequency in the population? Differences in the direction of preferences in natural populations is part of the original inspiration for the model and we have added the bowerbird reference mentioned here to lines 62. Our model presents a plausible mechanism by which such differences in preference direction could develop (which would be analogous to switching from a preference of TA1 to TA2). The example of swordtail species is intriguing but we did not add it here, as our focus is intraspecific (though we note in 399 that our idea also has implications for interactions among heterospecifics in hybrid zones.

6. line 365 do we really need the term "phenogenotype"? There is a well established framework, employed by some of the authors of this paper, for talking about and modeling phenotypes that are shaped by genotype and environment. We can talk about different possible social effects on preference without calling them "alleles", which is unnecessarily confusing since preferences are evolving.

**Author response**: The term “phenogenotype” is well established in gene-culture coevolutionary theory and has been used heavily by the authors of the current paper in discussing similar models. We are not aware of another way to refer to the joint “storage” in an individual of cultural and genetic information, or at least no way so succinct (“phenotype” would not work since in our case females do not express the phenotype at the TA and TB loci). We did get rid of the terms “alleles” and “loci” as shorthand for cultural traits, in response to this comment and a similar one from reviewer 1. We now use “cultural trait variant” and “cultural trait” instead.

7. line 339. "choosers" and "courters" versus "females" and "males". It might facilitate comparison across the literature if terminology never changed, but on the other hand science and society have been pushed forward by changes in unnecessarily gendered language. For this particular model, the classic paradigm of coy females and promiscuous males is certainly the most tractable and interesting way to approach it, but it's hard to see how this model doesn't apply to male-limited systems where males are the choosers.

**Author response:** We agree that this model certainly applies to male-limited systems where males are the choosers, and added a sentence to highlight that in lines 434-435.

8. line 426 and 497. Age affects a ton of things in real populations. Old females are copied more than younger females, and old females rely less on public information (at least in guppies). Age-related changes and even reversals in preference (e.g. bowerbirds above) are not unusual.  Female fecundity scales positively with age in many systems, as does male ornamentation and reproductive success. Given the importance of age structure to the dynamics of this model, this is worth mentioning.

**Author response**: Interestingly, in a meta-analysis of mate choice copying we previously found no evidence that older females were copied more than younger females (though younger females were indeed more likely to copy choices of older demonstrators, consistent with our model). These results are in Jones and DuVal 2017 Frontiers in Ecology and Evolution [doi: 10.3389/fevo.2019.00390. ]. Increased male ornamentation with age is an intriguing phenomenon with implications for how Inferred Attractiveness might act in natural populations, as we mention in lines 540-544.

9. line 519 I am a bit befuddled as to how the renormalization of trait distributions was actually accomplished…maybe a supplementary figure would be helpful for this if I'm not the only one.  I appreciate the importance of removing stochastic changes in trait frequencies to isolate the effects of preference evolution, but these stochastic changes…which by themselves change rare-male and copying dynamics…might change the game somehow, perhaps leading to fixation much more often than in the current model. 

**Author response:** Thanks for pointing out that this need clarification. We have added a more thorough description in lines 648-651.

Gil Rosenthal


Reviewer #3:

This paper presents an interesting and novel hypothesis of mate choice, developed as both a verbal and simulation model. "Inferred attractiveness" is a hypothesis based on mate choice copying and category learning that can explain fluctuations in preferences and sexual traits over time and contexts. I am excited to see this idea become further developed and tested. Suggestions for improvement of the manuscript follow, including major points first and then minor points line by line:

1. This hypothesis appears to be based on research in the cognitive sciences distinguishing classification and inference learning. These concepts should be explained in some detail—fleshing out the evidence and conceptual framework in references 36-38 so readers understand the empirical basis for the hypothesis; as written it does not seem as strongly justified as it could. I would recommend this come directly after the paragraph in which these references are introduced.

**Author response:** We have added additional information for each of the three assumptions noted in this paragraph. As part of that, we revised our choice of references to more specifically support the very general and widely supported statement that distinctive features are used to identify category membership, and also linked this third assumption more explicitly to discrimination learning. The papers focusing on classification vs inference learning are not as relevant here, but are now cited in lines 148, and 415-419 as examples of detailed analyses of category learning in humans.

2. Along the same lines, it is not clear why the observing chooser should always choose the most rare phenotype that distinguishes the category "chosen mate" - further empirical or logical bases for this prediction would help convince the reader.

**Author response:** Relative rarity is a key component of our model in that we assume that rarity corresponds to distinctiveness. We added support for this assumption, including more clear explanation of possible underlying mechanisms, in lines 146-154, but acknowledge in our exploration of unequal sexual selection (and sensory bias; lines 253-256) that some types of traits may be more salient than others. In that case, females disproportionately attend to some traits, but we still assume sensitivity to rarity of the alleles in that trait.

To be clear, we do want to note that while frequency of trait variants are used to establish female preferences in our model, females do not always end up choosing the most rare phenotype of males chosen by others. This is for several reasons. Most importantly, when females observe other choosing males that have the most common variant of both trait types, they will form a preference for the relatively rare of those common types. Furthermore, expressed mate choice is stochastically determined by the strength of sexual selection by mate choice (alpha) which is the likelihood a female will mate with a male that has one variant instead of the other, given that she encounters both. She does not always encounter both, and sometimes she still chooses the less preferred of the two options she considers. This is why patterns vary across values of alpha in Figures 1, 3, and 4.

3. Authors should speculate on the phylogenetic breadth of this mechanism. How many different species have been tested for this kind of category learning? Do the authors suppose this mechanism is as relevant for spiders and fish as it is for birds and people?

**Author response:** The reviewed draft noted the taxonomic breadth of Inferred Attractiveness at the end of the manuscript (lines 395-397). We have left that sentence in place, and added a sentence about taxonomic generality of the underlying assumptions of the mechanism in lines 146-148.

4. L75-78—this sentence, and throughout, reads as if there is no innate or otherwise learned preference that "battles" with the inference. Please speculate as to how they might interact - or is it supposed that the "chooser" (Fig. 1a) is simply basing their preferences on what they learned, and so on, with no original preferences, only copies and inference?

**Author response:** We agree that realized effects of the proposed mechanism could and probably will be tempered by innate preferences. In particular, we expect that these innate preferences will be the product of differences in the sensory systems of animals, and have added new text in lines 409-412 that more thoroughly address this point, including reference to the complex ways that innate and genetic preferences can interact.

5. L93—I don't think the authors mean "brighter" here—the chosen male looks less bright (more saturated red) than the unchosen.

**Author response:** We have replaced the word “brighter” with “more saturated”. (line 85)

6. L128-130—This sentence was hard to parse. "the trait of the chosen male that has the rarest" - is it the most rare allele of the trait, or the male with the rarest trait?

**Author response:** Change made to improve clarity.

7. L156—Lines have no color in this figure.

**Author response:** Change made, removing reference to line color.

8. L191-193—This sentence was hard to parse. What is consistent with what? I was similarly confused by 198-199. I'm not sure what represents a consistent pairing of facts.

**Author response:** Rephrased to improve clarity.

9. L199-201—Again confused. I think this might help: "Weak sexual selection on one of two traits WHEN VIABILITY SELECTION ON THAT TRAIT IS STRONG results in loss of trait variation AT THAT LOCUS, regardless of the strength of sexual (or viability?) selection on the other trait."

**Author response:** Revised for clarity, to read: “When viability selection is strong, weak sexual selection on one of two traits results in loss of trait variation at that locus, regardless of the strength of sexual selection on the other trait (upper portions of Fig. 3b.i, 3b.iii).” (lines 256-258)

10. Figure 3 - the levels of selection in the gray boxes are the same for a and b (ie constant sexual selection).

**Author response:** Yes, this is the case. We have added text to clarify this situation, also noted by reviewer 1.

11. L241-242—Phrasing is awkward—suggest "In the case of preference for novelty, preference extremes are observed only in large groups."

**Author response:** changes made throughout this paragraph to increase clarity.

L264—specify that "the 'selectively' favored genetic trait variant" is favored by viability or natural selection.

**Author response:** Change made to clarify that this refers to viability selection.

L324-327—this sentence did not make sense to me, not sure how to suggest a fix.

**Author response:** revised. New text is lines 406-415.

L415—I could have missed something, but what is "j mod"?

**Author response:** “mod” refers to the modulo operator. It is now defined in line 527. We have used it to enable us to write our equations very compactly.

__________________________________________________
In compliance with data protection regulations, you may request that we remove your personal registration details at any time.  (Use the following URL: [https://urldefense.com/v3/__https://www.editorialmanager.com/pbiology/login.asp?a=r__;!!PhOWcWs!1_C9QumoKrkkYS22F-v0qeUglSxuFsZPdmNKEdNexHk5B9nuTuL11hVz3PlUoXrkK9SyTvAWdWlLhizxwxk$](https://urldefense.com/v3/__https:/www.editorialmanager.com/pbiology/login.asp?a=r__;!!PhOWcWs!1_C9QumoKrkkYS22F-v0qeUglSxuFsZPdmNKEdNexHk5B9nuTuL11hVz3PlUoXrkK9SyTvAWdWlLhizxwxk$) ). Please contact the publication office if you have any questions.
